# FBX-DMEP suppresses hepatocellular carcinoma through tubulin-associated mitotic dysregulation

**DOI:** 10.1515/jtim-2026-0073

**Published:** 2026-08-24

**Authors:** Runhua Liu, Hongmei Li, Wei Zhao, Jianjiang Zhong, Yajie Tang

**Affiliations:** State Key Laboratory of Microbial Technology, Shandong University, Qingdao, Shandong Province, China; State Key Laboratory of Genetics and Development of Complex Phenotypes, College of Biotechnology, Fudan University, Shanghai, China; State Key Laboratory of Microbial Metabolism, Joint International Research Laboratory of Metabolic and Developmental Sciences, and School of Life Sciences and Biotechnology, Shanghai Jiao Tong University, Shanghai, China

## To the editor

Hepatocellular carcinoma (HCC) is the most prevalent primary hepatic malignancy and a leading cause of cancer-related mortality worldwide, with persistently limited systemic therapeutic options.^[1]^ Gene expression profiling allowed for identifying microtubules as a key therapeutic vulnerability in HCC.^[2]^ FBX-DMEP, originally reported as a topoisomerase II inhibitor,^[3]^ was found in this study to exhibit microtubule-associated activity. FBX-DMEP demonstrates potent cytotoxicity in hepatocellular carcinoma, robust antitumor efficacy in patient-derived organoid (PDO) and cell-derived xenograft (CDX) models, and an encouraging safety profile in preclinical studies, with no clinically relevant body weight loss or histopathological evidence of major organ toxicity. Taken together, these findings position FBX-DMEP as a promising candidate for translational development in HCC therapy.

**Figure 1:**
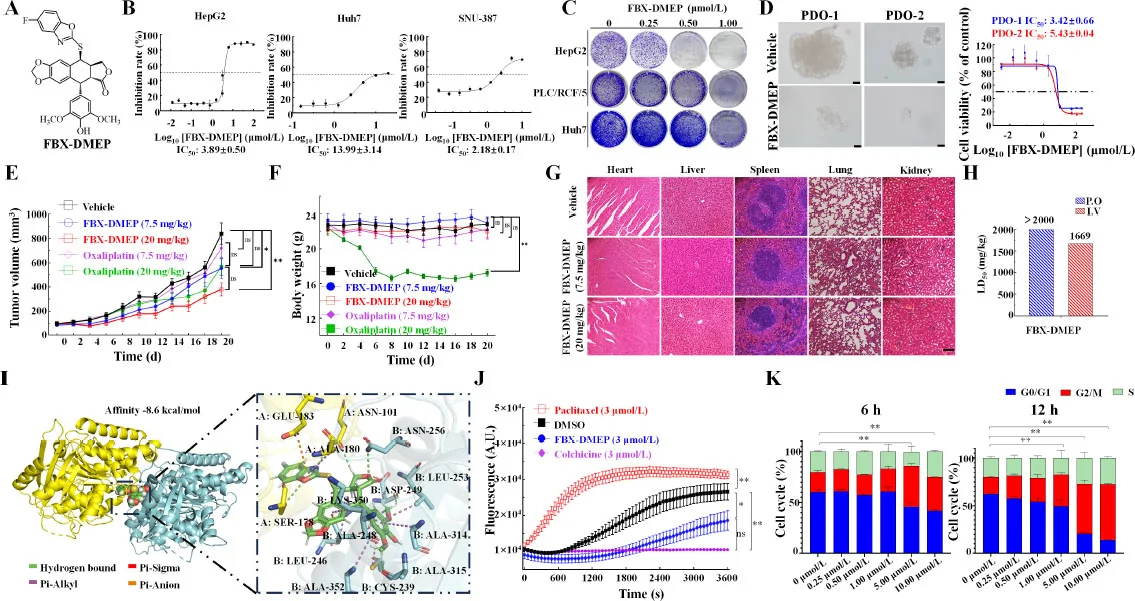
FBX-DMEP exhibits antitumor activity and microtubule-associated effects in HCC. (A) X-ray crystal structure of FBX-DMEP. (B) Dose-response analysis and IC_50_ determination in HCC cell lines (HepG2, Huh7, SNU-387). Data are presented as mean ± SEM from three independent experiments (*n* = 3). (C) Representative colony formation images and quantification in HepG2, PLC/PRF/5, and Huh7 cells following FBX-DMEP treatment. (D) Left: Growth inhibition of treatment-naïve patient-derived organoids (PDO-1 and PDO-2) treated with 200 μmol/L FBX-DMEP for 7 days. Right: Dose-response curves and IC values (mean ± SEM, *n* = 3) derived from 7-day FBX-DMEP treatment. (E) Tumor growth curves in the HepG2 CDX model. Mice were treated with vehicle control, FBX-DMEP (7.5 or 20 mg/kg *i.v*. every 2 days), or oxaliplatin (7.5 or 20 mg/kg). *n* = 6 per group. Vehicle *vs*. FBX-DMEP 7.5 mg/kg, *P* = 0.0404; vehicle *vs*. FBX-DMEP 20 mg/kg, *P* = 0.0007; vehicle *vs*. oxaliplatin 7.5 mg/kg, *P* = 0.6389; vehicle *vs*. oxaliplatin 20 mg/kg, *P* = 0.0694. (F) Body weight changes during the 20-day treatment period. Vehicle *vs*. FBX-DMEP 7.5 mg/kg, *P* = 0.9998; Vehicle *vs*. FBX-DMEP 20 mg/kg, *P* = 0.9199; oxaliplatin 7.5 mg/kg, *P* = 0.9692; vehicle *vs*. oxaliplatin 20 mg/kg, *P* = 0.0001. (G) Representative H&E staining of heart, liver, spleen, lung, and kidney from FBX-DMEP-treated mice (20 mg/kg) and vehicle controls. Scale bars: 100 μm. (H) Acute oral and intravenous LD_50_ values in Kunming mice (oral: > 2000 mg/kg; *i.v*.: 1669 mg/kg; 95%CI: 1122–2483 mg/kg); *n* = 10, 14 d. (I) Molecular docking analysis of FBX-DMEP within the colchicine-binding pocket of tubulin (PDB: 6XER), showing predicted interactions with α- and β-tubulin residues. (J) Tubulin polymerization was assessed *in vitro* using a fluorescence-based assay kit with purified porcine tubulin (BK011P, cytoskeleton). Fluorescence intensity was monitored over time after treatment with FBX-DMEP (3 μmol/L) and compared with paclitaxel (3 μmol/L), colchicine (3 μmol/L), and DMSO controls. AUC-based quantification (*n* = 4) showed significant differences between DMSO and paclitaxel (*P* = 0.0006), colchicine (*P* = 0.0037), and FBX-DMEP (*P* = 0.0120), whereas no significant difference was observed between FBX-DMEP and colchicine (*P* = 0.4524). (K) Cell cycle analysis of HepG2 cells treated with FBX-DMEP at 0.25, 0.5, 1.0, 5.0, and 10 μmol/L or with vehicle control (DMSO) for 6 h (left) or 12 h (right). **P < 0.01 compared with vehicle control at 6 h and 12 h. Statistical analyses were performed using one-way ANOVA with Tukey's post-hoc test for multiple comparisons. ns = not significant, *P < 0.05, **P < 0.01 vs. vehicle/DMSO control.

X-ray crystallographic analysis confirmed the chemical identity of FBX-DMEP as a DMEP-based derivative (Supplementary Figure S1 and Figure 1A). *In vitro* pharmacological profiling revealed broad-spectrum cytotoxicity against 12 cancer cell lines (Supplementary Table S1), while exhibiting minimal toxicity toward three non-transformed human cell lines-HL7702 (hepatic), HEK293T (renal), and MRC-5 (lung fibroblasts)-with IC_50_ values exceeding 200 μmol/L in all cases. Among HCC models, FBX-DMEP displayed consistent potency: IC_50_ values were 3.89 ± 0.50 μmol/L (HepG2), 13.99 μmol/L ± 3.14 μmol/L (Huh7), and 2.18 μmol/L ± 0.17 μmol/L (SNU-387) (Figure 1B). Clonogenic survival was impaired in HepG2, PLC/PRF/5, and Huh7 cells following FBX-DMEP treatment (Figure 1C). Two treatment-naïve PDO models (referred to as PDO-1 and PDO-2) were established. After 7 days of continuous exposure, FBX-DMEP induced dose-dependent reductions in organoid volume and viability, yielding CellTiter-Glo™-based IC_50_ values of 3.42 μmol/L ± 0.66 μmol/L (PDO-1) and 5.43 μmol/L ± 0.04 μmol/L (PDO-2) (Figure 1D).

We further evaluated *in vivo* antitumor efficacy of FBX-DMEP using a HepG2-derived subcutaneous xenograft model in immunocompromised male BALB/c nude mice. Upon tumor establishment (mean volume: 80–100 mm^3^), animals were randomized into five treatment groups (*n* = 6 per group) and administered intravenous doses every 48 h for 20 days: vehicle control (Cremophor EL vehicle), FBX-DMEP (7.5 or 20 mg/kg), and oxaliplatin (7.5 or 20 mg/kg). The doses of FBX-DMEP were selected based on preliminary tolerability studies, and oxaliplatin was administered at matched doses to allow head-to-head comparison under the same dosing conditions. FBX-DMEP treatment dose-dependently inhibited tumor growth, achieving 34% and 54% suppression at 7.5 and 20 mg/kg, respectively. No statistically significant difference in end-of-study tumor volume was observed between FBX-DMEP at 20 mg/kg and oxaliplatin at the same dose (Figure 1E). Notably, body weight remained stable throughout the study in all FBX-DMEP-treated cohorts, whereas mice receiving high-dose oxaliplatin (20 mg/kg) exhibited significant weight loss (> 20%) (Figure 1F). Hematoxylin and eosin (H&E) staining of heart, liver, spleen, lung, and kidney tissues revealed no treatment-associated morphological abnormalities (Figure 1G). Acute toxicity assessment in Kunming mice indicated favorable tolerability, with the oral LD_50_ exceeding 2000 mg/kg and the intravenous LD_50_ being 1669 mg/kg (95% CI: 1122–2483 mg/kg) (Figure 1H).

To elucidate the underlying molecular mechanisms, RNA sequencing (RNA-seq) was performed on HepG2 cells treated with FBX-DMEP or vehicle control (DMSO).

Differential expression analysis identified 494 significantly dysregulated genes (|fold change| ≥ 2, *P* < 0.05; Supplementary Figure S2A, Supplementary Tables S2–S4). KEGG pathway enrichment analysis revealed significant enrichment in microtubule-associated processes, most notably the cell cycle pathway (Supplementary Figure S2B). Comparative transcriptomic profiling comprising 93 upregulated and 150 downregulated genes was further interrogated using the Connectivity Map (CMAP) database.^[4]^ Strikingly, the FBX-DMEP signature showed high similarity to established tubulin-targeting agents, including vincristine and vinblastine (Supplementary Table S5). Consistent with these findings, gene set enrichment analysis (GSEA) using Hallmark gene sets demonstrated significant enrichment for “GOBP MITOTIC CELL CYCLE ARREST” and “GOBP ESTABLISHMENT OF MITOTIC SPINDLE ORIENTATION” (Figure S2C-S2D), supporting a functional role for FBX-DMEP in disrupting mitotic spindle architecture and perturbing cell cycle checkpoint control. Because cmap (connectivity map) similarity may reflect shared cytotoxic or cell cycle-related responses rather than specific target engagement, we further investigated the potential involvement of tubulin in subsequent experiments. Molecular docking simulations performed using the colchicine-bound tubulin structure (PDB: 6XER) predicted that FBX-DMEP may occupy the colchicine-binding pocket (-8.16 kcal/mol), with potential hydrogen bonds involving α-tubulin residues Asn101 and Ser178, and β-tubulin residues Asn256, Ala315, Asp249, alongside extensive hydrophobic interactions including π-anion, π-sigma, and π-alkyl contacts, that collectively stabilize ligand binding (Figure 1I). The experimentally resolved colchicine-tubulin interaction pattern from 6XER was further analyzed as a reference and shown in Figure S3. Further functional validation showed that FBX-DMEP significantly inhibited tubulin polymerization in a manner similar to the known microtubule inhibitor colchicine (Figure 1J). Collectively, these biochemical and computational results support tubulin engagement by FBX-DMEP and provide a structural hypothesis that FBX-DMEP may exert part of its anticancer activity through interaction with the colchicine-binding site of tubulin. However, the docking-derived residue-level interactions are predicted interactions and require further validation by orthogonal biophysical and structural approaches.

Consistent with tubulin inhibition, FBX-DMEP induced robust, dose- and time-dependent G_2_/M phase arrest in HepG2 cells. Flow cytometric analysis showed an increase in the G_2_/M population from ~20% in untreated controls to 40.1% following 6-hour treatment with 5 μmol/L FBX-DMEP and to 58.7% after 12-hour exposure to 10 μmol/L FBX-DMEP. This arrest coincided with elevated protein levels of mitotic markers MPM-2 and cyclin B (Figure 1K, Supplementary Figure S4). Moreover, FBX-DMEP triggered progressive apoptosis in a time-dependent manner: the total apoptotic fraction rose from 9.0 % at baseline (0 h) to 27.9 % at 24 h and 58.0 % at 48 h. Western blotting confirmed activation of the intrinsic apoptotic cascade, evidenced by increased cleavage of PARP and caspase-3, upregulation of pro-apoptotic Bax, and concomitant downregulation of anti-apoptotic Bcl-2 (Supplementary Figure S5).

FBX-DMEP is a rationally designed DMEP-based derivative that has been previously reported to possess topoisomerase II inhibitory activity. In the present study, we identify its microtubule-associated activity as a potential mechanism underlying its anticancer effects. Therefore, it holds promise as a novel, streamlined chemotherapeutic strategy for HCC. Nevertheless, several critical translational gaps remain. First, although molecular docking suggested that FBX-DMEP may occupy the colchicine-binding pocket of tubulin, colchicine was not redocked to tubulin in the present study to determine whether the docking protocol could reproduce the experimentally resolved colchicine-binding conformation. Direct target engagement must be rigorously validated using orthogonal biophysical approaches including high-resolution structural biology and quantitative binding assays. Second, although preliminary safety assessments indicate a favorable tolerability profile, comprehensive systemic toxicity evaluation including repeat-dose toxicokinetic and organ-specific histopathological analyses is warranted prior to clinical advancement. At present, only tubulin-related activity has been experimentally validated in this study, while the involvement of topoisomerase II remains unconfirmed and requires further investigation.

In summary, this study comprehensively characterized FBX-DMEP as a potential small-molecule agent that inhibits tubulin. Its potent anti-HCC activity was consistently demonstrated across *in vitro* models, patient-derived tumor organoids, and *in vivo* xenograft systems, coupled with a low off-target toxicity burden. These findings position FBX-DMEP as a promising preclinical candidate for the treatment of hepatocellular carcinoma.

## Supplementary Information

Supplementary materials are only available at the official site of the journal (www.intern-med.com).

## Supplementary Material

Supplementary Material Details
