# Supplementary material for: FBX-DMEP suppresses hepatocellular carcinoma through tubulin-associated mitotic dysregulation
Source: J Transl Int Med. 2026 Aug 24;14(4):650–3. doi: 10.1515/jtim-2026-0073 (PMC13509147; doi:10.1515/jtim-2026-0073)
Supplement: Supplementary file 1 [file jtim-2026-0073_sm.pdf]

Supplementary information

FBX-DMEP suppresses hepatocellular carcinoma through tubulin-associated mitotic dysregulation

Runhua Liu[1], Hongmei Li[1], Wei Zhao[1], Jianjiang Zhong[3], Yajie Tang[1,2]

[1]State Key Laboratory of Microbial Technology, Shandong University, Qingdao 266237, Shandong Province, China

[2]State Key Laboratory of Genetics and Development of Complex Phenotypes, College of Biotechnology, Fudan University, Shanghai 200433, China

[3]State Key Laboratory of Microbial Metabolism, Joint International Research Laboratory of Metabolic and Developmental Sciences, and School of Life Sciences and Biotechnology, Shanghai Jiao Tong University, Shanghai 200240, China

Address for Correspondence: Yajie Tang, State Key Laboratory of Genetics and Development of Complex Phenotypes, College of Biotechnology, Fudan University, Shanghai 200433, China. Email: yajietang@fudan.edu.cn; Jianjiang Zhong, State Key Laboratory of Microbial Metabolism, Joint International Research Laboratory of Metabolic and Developmental Sciences, and School of Life Sciences and Biotechnology, Shanghai Jiao Tong University, Shanghai 200240, China. Email: jjzhong@sjtu.edu.cn; Wei Zhao, State Key Laboratory of Microbial Technology, Shandong University, Qingdao 266237, Shandong Province, China. Email: weizhao2026@hbut.edu.cn

Supplementary Table S1. Half maximal inhibitory concentration (IC_{50}) values of FBX-DMEP against a panel of human cell lines. Data are presented as mean ± SEM from three independent experiments (*n* = 3), each performed in triplicate.

Tissue Origin	Cell Line	IC_{50} (μM)
Liver	HepG2	3.89±0.50
	Huh 7	13.99±3.14
	SNU-387	2.18±0.17
Lymphoma	SU-DHL-4	2.09±0.16
Leukemia	Jurkat	3.42±2.07
	K562	5.25±1.95
Lung	A549	7.86±1.59
Stomach	HGC27	21.52±1.28
Cervical	Hela	4.72±0.32
Melanoma	A375	77.9±1.57
Prostate	DU145	29.7±2.21
Colon	HT-29	8.56±5.73
Normal cell lines	HL7702	>200
	HEK293T	>200
	MRC-5	>200

Supplementary Table S2. 494 differentially expressed genes in HepG2 cells after 48 hours of treatment with 2.5 μM FBX-DMEP

Gene ID	Gene Symbol	Type	log2 (FBX-DMEP /DMSO)	Qvalue (FBX-DMEP /DMSO)	Pvalue (FBX-DMEP /DMSO)
100526832	'PHOSPHO2-KLHL23'	mRNA	-6.43	0.00	0.00
145781	'GCOM1'	mRNA	-5.48	0.13	0.01
4056	'LTC4S'	mRNA	-5.07	0.30	0.03
56896	'DPYSL5'	mRNA	-4.59	0.24	0.02
101059918	'GOLGA8R'	mRNA	-4.46	0.21	0.02
84767	'TRIM51'	mRNA	-4.35	0.15	0.01
221409	'SPATS1'	mRNA	-4.22	0.29	0.03
79170	'PRR15L'	mRNA	-4.22	0.07	0.00
441381	'LRRC24'	mRNA	-4.11	0.29	0.03
9032	'TM4SF5'	mRNA	-4.10	0.37	0.05
84634	'KISS1R'	mRNA	-4.09	0.35	0.04
9378	'NRXN1'	mRNA	-4.09	0.16	0.01
23768	'FLRT2'	mRNA	-4.07	0.26	0.02
780776	'TVP23A'	mRNA	-3.90	0.22	0.02
360205	'PRAC2'	mRNA	-3.79	0.33	0.04
1780	'DYNC1I1'	mRNA	-3.68	0.34	0.04
342618	'SLFN14'	mRNA	-3.39	0.23	0.02
50832	'TAS2R4'	mRNA	-3.27	0.24	0.02
643669	'CCER2'	mRNA	-3.24	0.33	0.04
388564	'TMEM238'	mRNA	-3.22	0.18	0.01
25803	'SPDEF'	mRNA	-3.18	0.06	0.00

8577	'TMEFF1'	mRNA	-3.14	0.11	0.00
1890	'TYMP'	mRNA	-3.12	0.21	0.02
8294	'H4C9'	mRNA	-2.86	0.33	0.04
63827	'BCAN'	mRNA	-2.76	0.06	0.00
2657	'GDF1'	mRNA	-2.75	0.30	0.03
100996842	'LOC100996842'	mRNA	-2.73	0.03	0.00
11309	'SLCO2B1'	mRNA	-2.63	0.35	0.04
4917	'NTN3'	mRNA	-2.61	0.17	0.01
57165	'GJC2'	mRNA	-2.61	0.14	0.01
4902	'NRTN'	mRNA	-2.58	0.08	0.00
1299	'COL9A3'	mRNA	-2.54	0.31	0.03
79927	'FAM110D'	mRNA	-2.48	0.29	0.03
79946	'C10orf95'	mRNA	-2.47	0.06	0.00
196740	'VSTM4'	mRNA	-2.47	0.28	0.03
728577	'CNTNAP3B'	mRNA	-2.46	0.09	0.00
642515	'PRRT1B'	mRNA	-2.42	0.28	0.03
140825	'NEURL2'	mRNA	-2.42	0.25	0.02
2868	'GRK4'	mRNA	-2.41	0.18	0.01
3706	'ITPKA'	mRNA	-2.40	0.02	0.00
3718	'JAK3'	mRNA	-2.39	0.26	0.02
7153	'TOP2A'	mRNA	-2.36	0.00	0.00
1137	'CHRNA4'	mRNA	-2.36	0.20	0.01
54577	'UGT1A7'	mRNA	-2.34	0.31	0.03
10023	'FRAT1'	mRNA	-2.32	0.06	0.00
83729	'INHBE'	mRNA	-2.30	0.12	0.01
9241	'NOG'	mRNA	-2.27	0.25	0.02
54492	'NEURL1B'	mRNA	-2.26	0.00	0.00

89870	'TRIM15'	mRNA	-2.26	0.27	0.03
115650	'TNFRSF13C'	mRNA	-2.24	0.11	0.00
11094	'CACFD1'	mRNA	-2.21	0.04	0.00
23204	'ARL6IP1'	mRNA	-2.18	0.00	0.00
10732	'TCFL5'	mRNA	-2.16	0.30	0.03
83259	'PCDH11Y'	mRNA	-2.14	0.31	0.03
1825	'DSC3'	mRNA	-2.13	0.22	0.02
728116	'ZBTB8B'	mRNA	-2.13	0.31	0.03
5648	'MASP1'	mRNA	-2.13	0.22	0.02
221914	'GPC2'	mRNA	-2.12	0.16	0.01
161882	'ZFPM1'	mRNA	-2.06	0.04	0.00
728492	'SERF1B'	mRNA	-2.04	0.32	0.04
55897	'MESP1'	mRNA	-2.03	0.35	0.04
646960	'PRSS56'	mRNA	-2.02	0.22	0.02
79788	'ZNF665'	mRNA	-2.00	0.32	0.04
94234	'FOXQ1'	mRNA	-1.99	0.24	0.02
90113	'VWA5B2'	mRNA	-1.99	0.30	0.03
56936	'CCDC177'	mRNA	-1.99	0.05	0.00
83481	'EPPK1'	mRNA	-1.96	0.29	0.03
401720	'FIGNL2'	mRNA	-1.95	0.09	0.00
84278	'MFSD14C'	mRNA	-1.94	0.24	0.02
1277	'COL1A1'	mRNA	-1.93	0.34	0.04
3727	'JUND'	mRNA	-1.93	0.01	0.00
100132074	'FOXO6'	mRNA	-1.92	0.11	0.00
201255	'LRRC45'	mRNA	-1.92	0.27	0.03
687	'KLF9'	mRNA	-1.92	0.18	0.01
5372	'PMM1'	mRNA	-1.91	0.37	0.05

388323	'GLTPD2'	mRNA	-1.88	0.29	0.03
6752	'SSTR2'	mRNA	-1.86	0.20	0.01
253559	'CADM2'	mRNA	-1.86	0.36	0.05
84968	'PNMA6A'	mRNA	-1.85	0.17	0.01
79782	'LRRC31'	mRNA	-1.83	0.03	0.00
389692	'MAFA'	mRNA	-1.81	0.28	0.03
1628	'DBP'	mRNA	-1.81	0.06	0.00
155382	'VPS37D'	mRNA	-1.79	0.33	0.04
389816	'LRRC26'	mRNA	-1.79	0.03	0.00
199699	'DAND5'	mRNA	-1.79	0.31	0.03
23101	'MCF2L2'	mRNA	-1.78	0.22	0.02
3795	'KHK'	mRNA	-1.78	0.02	0.00
79842	'ZBTB3'	mRNA	-1.77	0.01	0.00
5334	'PLCL1'	mRNA	-1.77	0.01	0.00
90427	'BMF'	mRNA	-1.75	0.05	0.00
1028	'CDKN1C'	mRNA	-1.75	0.01	0.00
25794	'FSCN2'	mRNA	-1.74	0.28	0.03
79632	'FAM184A'	mRNA	-1.73	0.30	0.03
91828	'EXOC3L4'	mRNA	-1.72	0.29	0.03
10675	'CSPG5'	mRNA	-1.72	0.00	0.00
79772	'MCTP1'	mRNA	-1.71	0.02	0.00
255783	'INAFM1'	mRNA	-1.71	0.21	0.02
55450	'CAMK2N1'	mRNA	-1.70	0.03	0.00
53342	'IL17D'	mRNA	-1.69	0.26	0.02
983	'CDK1'	mRNA	-1.68	0.00	0.00
643680	'MS4A4E'	mRNA	-1.68	0.31	0.03
4130	'MAP1A'	mRNA	-1.67	0.00	0.00

84922	'FIZ1'	mRNA	-1.67	0.01	0.00
2306	'FOXD2'	mRNA	-1.67	0.22	0.02
155185	'AMZ1'	mRNA	-1.66	0.03	0.00
11065	'UBE2C'	mRNA	-1.66	0.00	0.00
353376	'TICAM2'	mRNA	-1.65	0.30	0.03
83882	'TSPAN10'	mRNA	-1.65	0.20	0.01
3110	'MNX1'	mRNA	-1.64	0.17	0.01
255374	'MBLAC1'	mRNA	-1.63	0.34	0.04
6094	'ROM1'	mRNA	-1.63	0.32	0.04
254552	'NUDT8'	mRNA	-1.63	0.17	0.01
379	'ARL4D'	mRNA	-1.60	0.04	0.00
401097	'C3orf80'	mRNA	-1.59	0.11	0.00
57622	'LRFN1'	mRNA	-1.59	0.00	0.00
91179	'SCARF2'	mRNA	-1.59	0.18	0.01
100507588	'TGFBR3L'	mRNA	-1.58	0.13	0.01
55512	'SMPD3'	mRNA	-1.57	0.32	0.04
79002	'TRIR'	mRNA	-1.57	0.01	0.00
90673	'PPP1R3E'	mRNA	-1.57	0.08	0.00
135152	'B3GAT2'	mRNA	-1.56	0.32	0.04
257	'ALX3'	mRNA	-1.55	0.01	0.00
376497	'SLC27A1'	mRNA	-1.54	0.02	0.00
162494	'RHBDL3'	mRNA	-1.54	0.15	0.01
8364	'H4C3'	mRNA	-1.53	0.25	0.02
114783	'LMTK3'	mRNA	-1.53	0.24	0.02
388403	'YPEL2'	mRNA	-1.53	0.01	0.00
388963	'C2orf81'	mRNA	-1.53	0.23	0.02
6659	'SOX4'	mRNA	-1.53	0.00	0.00

56413	'LTB4R2'	mRNA	-1.52	0.18	0.01
93145	'OLFM2'	mRNA	-1.50	0.22	0.02
23232	'TBC1D12'	mRNA	-1.50	0.01	0.00
147687	'ZNF417'	mRNA	-1.49	0.03	0.00
27022	'FOXD3'	mRNA	-1.48	0.33	0.04
219699	'UNC5B'	mRNA	-1.48	0.02	0.00
80352	'RNF39'	mRNA	-1.48	0.27	0.02
3903	'LAIR1'	mRNA	-1.46	0.21	0.02
340152	'ZC3H12D'	mRNA	-1.45	0.29	0.03
200312	'RNF215'	mRNA	-1.45	0.21	0.02
64599	'GIGYF1'	mRNA	-1.45	0.01	0.00
79447	'PAGR1'	mRNA	-1.45	0.18	0.01
374920	'ZSWIM9'	mRNA	-1.44	0.22	0.02
7475	'WNT6'	mRNA	-1.44	0.23	0.02
220213	'OTUD1'	mRNA	-1.43	0.13	0.01
648791	'PPP1R3G'	mRNA	-1.43	0.13	0.01
110806298	'C17orf113'	mRNA	-1.42	0.24	0.02
51222	'ZNF219'	mRNA	-1.42	0.19	0.01
29775	'CARD10'	mRNA	-1.42	0.04	0.00
387787	'LIPT2'	mRNA	-1.40	0.17	0.01
7465	'WEE1'	mRNA	-1.40	0.00	0.00
5106	'PCK2'	mRNA	-1.40	0.20	0.01
10451	'VAV3'	mRNA	-1.39	0.00	0.00
117247	'SLC16A10'	mRNA	-1.39	0.17	0.01
90007	'MIDN'	mRNA	-1.39	0.00	0.00
7161	'TP73'	mRNA	-1.39	0.18	0.01
9028	'RHBDL1'	mRNA	-1.38	0.24	0.02

158055	'C9orf163'	mRNA	-1.38	0.28	0.03
58487	'CREBZF'	mRNA	-1.38	0.04	0.00
148252	'DIRAS1'	mRNA	-1.38	0.27	0.02
57134	'MAN1C1'	mRNA	-1.38	0.16	0.01
55147	'RBM23'	mRNA	-1.38	0.00	0.00
3838	'KPNA2'	mRNA	-1.38	0.02	0.00
63920	'ZBED8'	mRNA	-1.38	0.16	0.01
50509	'COL5A3'	mRNA	-1.37	0.33	0.04
5507	'PPP1R3C'	mRNA	-1.37	0.04	0.00
3868	'KRT16'	mRNA	-1.37	0.19	0.01
7018	'TF'	mRNA	-1.37	0.17	0.01
118460	'EXOSC6'	mRNA	-1.36	0.14	0.01
7428	'VHL'	mRNA	-1.36	0.17	0.01
5296	'PIK3R2'	mRNA	-1.36	0.00	0.00
3619	'INCENP'	mRNA	-1.36	0.00	0.00
2297	'FOXD1'	mRNA	-1.35	0.22	0.02
55374	'TMCO6'	mRNA	-1.34	0.21	0.01
9783	'RIMS3'	mRNA	-1.34	0.22	0.02
284023	'RNF227'	mRNA	-1.34	0.00	0.00
7049	'TGFBR3'	mRNA	-1.33	0.00	0.00
9435	'CHST2'	mRNA	-1.33	0.28	0.03
125170	'MIEF2'	mRNA	-1.33	0.10	0.00
10290	'SPEG'	mRNA	-1.33	0.10	0.00
54910	'SEMA4C'	mRNA	-1.32	0.06	0.00
283208	'P4HA3'	mRNA	-1.32	0.18	0.01
55344	'PLCXD1'	mRNA	-1.32	0.00	0.00
79798	'ARMC5'	mRNA	-1.32	0.21	0.01

92815	'H2AW'	mRNA	-1.31	0.37	0.05
55561	'CDC42BPG'	mRNA	-1.31	0.12	0.01
84705	'GTPBP3'	mRNA	-1.31	0.01	0.00
57547	'ZNF624'	mRNA	-1.31	0.21	0.01
23467	'NPTXR'	mRNA	-1.31	0.00	0.00
23385	'NCSTN'	mRNA	-1.31	0.01	0.00
899	'CCNF'	mRNA	-1.31	0.00	0.00
126133	'ALDH16A1'	mRNA	-1.31	0.15	0.01
57010	'CABP4'	mRNA	-1.31	0.17	0.01
57716	'PRX'	mRNA	-1.30	0.28	0.03
23178	'PASK'	mRNA	-1.30	0.01	0.00
84264	'HAGHL'	mRNA	-1.29	0.28	0.03
84932	'RAB2B'	mRNA	-1.29	0.00	0.00
4092	'SMAD7'	mRNA	-1.29	0.00	0.00
9826	'ARHGEF11'	mRNA	-1.29	0.00	0.00
349075	'ZNF713'	mRNA	-1.29	0.07	0.00
1870	'E2F2'	mRNA	-1.28	0.00	0.00
8325	'FZD8'	mRNA	-1.28	0.22	0.02
84787	'KMT5C'	mRNA	-1.28	0.05	0.00
8646	'CHRD'	mRNA	-1.28	0.26	0.02
4082	'MARCKS'	mRNA	-1.27	0.18	0.01
3216	'HOXB6'	mRNA	-1.27	0.28	0.03
89849	'ATG16L2'	mRNA	-1.27	0.21	0.02
11264	'PXMP4'	mRNA	-1.27	0.28	0.03
84915	'FAM222A'	mRNA	-1.27	0.11	0.00
2116	'ETV2'	mRNA	-1.26	0.35	0.04
2178	'FANCE'	mRNA	-1.26	0.07	0.00

389333	'PROB1'	mRNA	-1.25	0.28	0.03
57643	'ZSWIM5'	mRNA	-1.25	0.00	0.00
401265	'KLHL31'	mRNA	-1.25	0.26	0.02
1869	'E2F1'	mRNA	-1.25	0.01	0.00
9696	'CROCC'	mRNA	-1.25	0.26	0.02
138428	'PTRH1'	mRNA	-1.25	0.31	0.03
113115	'MTFR2'	mRNA	-1.25	0.05	0.00
90353	'CTU1'	mRNA	-1.25	0.35	0.04
54876	'DCAF16'	mRNA	-1.24	0.00	0.00
4162	'MCAM'	mRNA	-1.24	0.00	0.00
26271	'FBXO5'	mRNA	-1.24	0.00	0.00
359948	'IRF2BP2'	mRNA	-1.24	0.17	0.01
414919	'C8orf82'	mRNA	-1.23	0.31	0.03
285521	'COX18'	mRNA	-1.23	0.16	0.01
6456	'SH3GL2'	mRNA	-1.23	0.01	0.00
84875	'PARP10'	mRNA	-1.23	0.18	0.01
144699	'FBXL14'	mRNA	-1.23	0.16	0.01
3303	'HSPA1A'	mRNA	-1.23	0.09	0.00
10181	'RBM5'	mRNA	-1.23	0.00	0.00
388552	'BLOC1S3'	mRNA	-1.22	0.06	0.00
8535	'CBX4'	mRNA	-1.22	0.06	0.00
580	'BARD1'	mRNA	-1.22	0.00	0.00
10251	'SPRY3'	mRNA	-1.22	0.30	0.03
29904	'EEF2K'	mRNA	-1.21	0.00	0.00
2319	'FLOT2'	mRNA	-1.21	0.00	0.00
1316	'KLF6'	mRNA	-1.20	0.00	0.00
9592	'IER2'	mRNA	-1.20	0.31	0.03

2621	'GAS6'	mRNA	-1.20	0.22	0.02
100533105	'C8orf44-SGK3'	mRNA	-1.20	0.35	0.05
116028	'RMI2'	mRNA	-1.20	0.08	0.00
7374	'UNG'	mRNA	-1.19	0.00	0.00
114822	'RHPN1'	mRNA	-1.19	0.29	0.03
79037	'PVRIG'	mRNA	-1.19	0.35	0.04
57479	'PRR12'	mRNA	-1.19	0.00	0.00
1943	'EFNA2'	mRNA	-1.19	0.27	0.03
29968	'PSAT1'	mRNA	-1.18	0.00	0.00
90576	'ZNF799'	mRNA	-1.18	0.32	0.04
2030	'SLC29A1'	mRNA	-1.18	0.01	0.00
3209	'HOXA13'	mRNA	-1.18	0.00	0.00
41	'ASIC1'	mRNA	-1.18	0.00	0.00
254013	'ETFBKMT'	mRNA	-1.18	0.34	0.04
144455	'E2F7'	mRNA	-1.17	0.00	0.00
22859	'ADGRL1'	mRNA	-1.17	0.01	0.00
9451	'EIF2AK3'	mRNA	-1.17	0.00	0.00
9355	'LHX2'	mRNA	-1.17	0.21	0.01
399664	'MEX3D'	mRNA	-1.17	0.32	0.04
10347	'ABCA7'	mRNA	-1.17	0.31	0.03
729438	'CASTOR2'	mRNA	-1.17	0.02	0.00
248	'ALPI'	mRNA	-1.17	0.15	0.01
79803	'HPS6'	mRNA	-1.16	0.06	0.00
51337	'THEM6'	mRNA	-1.16	0.18	0.01
23456	'ABCB10'	mRNA	-1.16	0.11	0.00
340719	'NANOS1'	mRNA	-1.16	0.33	0.04
100131244	'ANKRD63'	mRNA	-1.16	0.35	0.04

1808	'DPYSL2'	mRNA	-1.16	0.00	0.00
5190	'PEX6'	mRNA	-1.16	0.30	0.03
126272	'EID2B'	mRNA	-1.16	0.32	0.04
23211	'ZC3H4'	mRNA	-1.15	0.00	0.00
51043	'ZBTB7B'	mRNA	-1.15	0.21	0.01
25840	'METTL7A'	mRNA	-1.15	0.15	0.01
2634	'GBP2'	mRNA	-1.14	0.28	0.03
9837	'GINS1'	mRNA	-1.14	0.00	0.00
29946	'SERTAD3'	mRNA	-1.14	0.00	0.00
5604	'MAP2K1'	mRNA	-1.14	0.30	0.03
374654	'KIF7'	mRNA	-1.14	0.17	0.01
51005	'AMDHD2'	mRNA	-1.13	0.27	0.02
100381270	'ZBED6'	mRNA	-1.13	0.00	0.00
128272	'ARHGEF19'	mRNA	-1.13	0.28	0.03
10882	'C1QL1'	mRNA	-1.13	0.34	0.04
9422	'ZNF264'	mRNA	-1.13	0.01	0.00
55160	'ARHGEF10L'	mRNA	-1.13	0.17	0.01
63971	'KIF13A'	mRNA	-1.13	0.00	0.00
140545	'RNF32'	mRNA	-1.13	0.33	0.04
100293516	'ZNF587B'	mRNA	-1.13	0.01	0.00
439921	'MXRA7'	mRNA	-1.13	0.34	0.04
80235	'PIGZ'	mRNA	-1.13	0.18	0.01
9894	'TELO2'	mRNA	-1.13	0.19	0.01
901	'CCNG2'	mRNA	-1.12	0.10	0.00
8425	'LTBP4'	mRNA	-1.12	0.25	0.02
10965	'ACOT2'	mRNA	-1.12	0.32	0.04
8877	'SPHK1'	mRNA	-1.12	0.28	0.03

7138	'TNNT1'	mRNA	-1.12	0.13	0.01
382	'ARF6'	mRNA	-1.12	0.07	0.00
151903	'CCDC12'	mRNA	-1.12	0.03	0.00
2021	'ENDOG'	mRNA	-1.12	0.37	0.05
727910	'TLCD2'	mRNA	-1.12	0.34	0.04
51282	'SCAND1'	mRNA	-1.12	0.34	0.04
5933	'RBL1'	mRNA	-1.11	0.00	0.00
10763	'NES'	mRNA	-1.11	0.07	0.00
201266	'SLC39A11'	mRNA	-1.11	0.13	0.01
6666	'SOX12'	mRNA	-1.11	0.31	0.03
26000	'TBC1D10B'	mRNA	-1.11	0.05	0.00
84687	'PPP1R9B'	mRNA	-1.11	0.02	0.00
8693	'GALNT4'	mRNA	-1.10	0.26	0.02
727800	'RNF208'	mRNA	-1.10	0.35	0.04
2188	'FANCF'	mRNA	-1.10	0.09	0.00
254102	'EHBP1L1'	mRNA	-1.10	0.24	0.02
54345	'SOX18'	mRNA	-1.10	0.32	0.04
4131	'MAP1B'	mRNA	-1.10	0.00	0.00
6751	'SSTR1'	mRNA	-1.10	0.00	0.00
284058	'KANSL1'	mRNA	-1.10	0.00	0.00
23401	'FRAT2'	mRNA	-1.10	0.30	0.03
26048	'ZNF500'	mRNA	-1.10	0.03	0.00
284004	'HEXD'	mRNA	-1.10	0.25	0.02
79873	'NUDT18'	mRNA	-1.10	0.34	0.04
8743	'TNFSF10'	mRNA	-1.09	0.25	0.02
573	'BAG1'	mRNA	-1.09	0.28	0.03
286257	'PAXX'	mRNA	-1.09	0.31	0.03

9672	'SDC3'	mRNA	-1.09	0.00	0.00
92979	'MARCHF9'	mRNA	-1.09	0.29	0.03
642475	'MROH6'	mRNA	-1.09	0.29	0.03
94122	'SYTL5'	mRNA	-1.08	0.31	0.03
10279	'PRSS16'	mRNA	-1.08	0.13	0.01
166824	'RASSF6'	mRNA	-1.08	0.29	0.03
151230	'KLHL23'	mRNA	-1.08	0.01	0.00
8771	'TNFRSF6B'	mRNA	-1.08	0.26	0.02
55367	'PIDD1'	mRNA	-1.08	0.19	0.01
898	'CCNE1'	mRNA	-1.08	0.00	0.00
90378	'SAMD1'	mRNA	-1.08	0.00	0.00
334	'APLP2'	mRNA	-1.08	0.00	0.00
6494	'SIPA1'	mRNA	-1.08	0.19	0.01
3217	'HOXB7'	mRNA	-1.08	0.05	0.00
56937	'PMEPA1'	mRNA	-1.07	0.00	0.00
1747	'DLX3'	mRNA	-1.07	0.07	0.00
29057	'FAM156A'	mRNA	-1.07	0.03	0.00
101928841	'LOC101928841'	mRNA	-1.07	0.21	0.02
10541	'ANP32B'	mRNA	-1.07	0.32	0.04
284069	'FAM171A2'	mRNA	-1.07	0.34	0.04
54058	'C21orf58'	mRNA	-1.07	0.14	0.01
9134	'CCNE2'	mRNA	-1.06	0.08	0.00
23468	'CBX5'	mRNA	-1.06	0.01	0.00
8408	'ULK1'	mRNA	-1.06	0.31	0.03
92092	'ZC3HAV1L'	mRNA	-1.06	0.20	0.01
54487	'DGCR8'	mRNA	-1.06	0.00	0.00
388228	'SBK1'	mRNA	-1.06	0.34	0.04

101060684	'NBPF26'	mRNA	-1.06	0.24	0.02
2109	'ETFB'	mRNA	-1.06	0.35	0.04
5413	'SEPTIN5'	mRNA	-1.06	0.10	0.00
81621	'KAZALD1'	mRNA	-1.06	0.21	0.02
1060	'CENPC'	mRNA	-1.06	0.04	0.00
79191	'IRX3'	mRNA	-1.06	0.23	0.02
79713	'IGFLR1'	mRNA	-1.05	0.13	0.01
10607	'TBL3'	mRNA	-1.05	0.21	0.02
338879	'RNASE10'	mRNA	-1.05	0.31	0.03
6941	'TCF19'	mRNA	-1.05	0.00	0.00
91748	'MIDEAS'	mRNA	-1.05	0.00	0.00
113146	'AHNAK2'	mRNA	-1.05	0.00	0.00
22920	'KIFAP3'	mRNA	-1.05	0.00	0.00
126792	'B3GALT6'	mRNA	-1.05	0.22	0.02
2444	'FRK'	mRNA	-1.05	0.06	0.00
727	'C5'	mRNA	-1.05	0.13	0.01
550631	'CCDC157'	mRNA	-1.04	0.26	0.02
116225	'ZMYND19'	mRNA	-1.04	0.35	0.04
55027	'HEATR3'	mRNA	-1.04	0.00	0.00
2619	'GAS1'	mRNA	-1.04	0.25	0.02
9781	'RNF144A'	mRNA	-1.04	0.21	0.02
84936	'ZFYVE19'	mRNA	-1.04	0.34	0.04
91433	'RCCD1'	mRNA	-1.04	0.22	0.02
4241	'MELTF'	mRNA	-1.04	0.23	0.02
10536	'P3H3'	mRNA	-1.04	0.37	0.05
84061	'MAGT1'	mRNA	-1.04	0.28	0.03
85451	'UNK'	mRNA	-1.04	0.00	0.00

6614	'SIGLEC1'	mRNA	-1.04	0.31	0.03
4172	'MCM3'	mRNA	-1.03	0.01	0.00
29781	'NCAPH2'	mRNA	-1.03	0.27	0.02
8208	'CHAF1B'	mRNA	-1.03	0.00	0.00
107983993	'UPK3BL2'	mRNA	-1.03	0.35	0.04
1852	'DUSP9'	mRNA	-1.03	0.33	0.04
390980	'ZNF805'	mRNA	-1.03	0.21	0.01
390874	'ONECUT3'	mRNA	-1.03	0.09	0.00
954	'ENTPD2'	mRNA	-1.02	0.23	0.02
56654	'NPDC1'	mRNA	-1.02	0.35	0.04
387509	'GPR153'	mRNA	-1.02	0.18	0.01
65988	'ZNF747'	mRNA	-1.02	0.05	0.00
283008	'NUTM2E'	mRNA	-1.02	0.37	0.05
79812	'MMRN2'	mRNA	-1.02	0.31	0.03
23403	'FBXO46'	mRNA	-1.02	0.03	0.00
8799	'PEX11B'	mRNA	-1.02	0.06	0.00
114787	'GPRIN1'	mRNA	-1.02	0.00	0.00
783	'CACNB2'	mRNA	-1.02	0.10	0.00
79966	'SCD5'	mRNA	-1.02	0.07	0.00
768211	'RELL1'	mRNA	-1.01	0.10	0.00
55719	'SLF2'	mRNA	-1.01	0.00	0.00
2837	'UTS2R'	mRNA	-1.01	0.30	0.03
348487	'FAM131C'	mRNA	-1.01	0.18	0.01
643988	'FNDC10'	mRNA	-1.01	0.10	0.00
3169	'FOXA1'	mRNA	-1.00	0.00	0.00
200205	'IBA57'	mRNA	-1.00	0.13	0.01
30846	'EHD2'	mRNA	-1.00	0.00	0.00

93185	'IGSF8'	mRNA	-1.00	0.28	0.03
1609	'DGKQ'	mRNA	-1.00	0.34	0.04
3229	'HOXC13'	mRNA	1.00	0.00	0.00
79844	'ZDHHC11'	mRNA	1.01	0.14	0.01
4281	'MID1'	mRNA	1.02	0.06	0.00
10627	'MYL12A'	mRNA	1.02	0.13	0.01
2274	'FHL2'	mRNA	1.02	0.01	0.00
4356	'MPP3'	mRNA	1.03	0.00	0.00
3383	'ICAM1'	mRNA	1.03	0.01	0.00
8347	'H2BC4'	mRNA	1.03	0.08	0.00
7099	'TLR4'	mRNA	1.04	0.17	0.01
9531	'BAG3'	mRNA	1.05	0.04	0.00
23117	'NPIPB3'	mRNA	1.06	0.00	0.00
115362	'GBP5'	mRNA	1.06	0.11	0.00
6790	'AURKA'	mRNA	1.07	0.05	0.00
55062	'WIPI1'	mRNA	1.07	0.04	0.00
390	'RND3'	mRNA	1.08	0.01	0.00
80149	'ZC3H12A'	mRNA	1.08	0.00	0.00
10024	'TROAP'	mRNA	1.09	0.00	0.00
652	'BMP4'	mRNA	1.10	0.08	0.00
3931	'LCAT'	mRNA	1.10	0.21	0.02
2152	'F3'	mRNA	1.11	0.06	0.00
4862	'NPAS2'	mRNA	1.11	0.05	0.00
10202	'DHRS2'	mRNA	1.11	0.24	0.02
2118	'ETV4'	mRNA	1.11	0.00	0.00
375791	'CYSRT1'	mRNA	1.12	0.01	0.00
26579	'MYEOV'	mRNA	1.13	0.35	0.04

152519	'NIPAL1'	mRNA	1.13	0.05	0.00
90417	'KNSTRN'	mRNA	1.15	0.11	0.00
100132247	'NPIPB5'	mRNA	1.17	0.01	0.00
102800317	'TPTEP2-CSNK1E'	mRNA	1.18	0.20	0.01
51330	'TNFRSF12A'	mRNA	1.18	0.03	0.00
1969	'EPHA2'	mRNA	1.18	0.00	0.00
56256	'SERTAD4'	mRNA	1.19	0.04	0.00
314	'AOC2'	mRNA	1.21	0.21	0.01
991	'CDC20'	mRNA	1.23	0.01	0.00
29091	'STXBP6'	mRNA	1.27	0.07	0.00
79628	'SH3TC2'	mRNA	1.27	0.10	0.00
8870	'IER3'	mRNA	1.28	0.00	0.00
3885	'KRT34'	mRNA	1.29	0.28	0.03
22822	'PHLDA1'	mRNA	1.30	0.04	0.00
1821	'DRP2'	mRNA	1.30	0.15	0.01
8341	'H2BC15'	mRNA	1.33	0.31	0.03
50617	'ATP6V0A4'	mRNA	1.33	0.06	0.00
2354	'FOSB'	mRNA	1.33	0.02	0.00
199800	'ADM5'	mRNA	1.34	0.01	0.00
5923	'RASGRF1'	mRNA	1.35	0.27	0.02
114769	'CARD16'	mRNA	1.37	0.08	0.00
10769	'PLK2'	mRNA	1.37	0.01	0.00
8676	'STX11'	mRNA	1.38	0.33	0.04
57393	'CLTRN'	mRNA	1.39	0.08	0.00
613037	'NPIPB13'	mRNA	1.47	0.23	0.02
1164	'CKS2'	mRNA	1.49	0.06	0.00
220001	'VWCE'	mRNA	1.52	0.29	0.03

84959	'UBASH3B'	mRNA	1.53	0.17	0.01
3017	'H2BC5'	mRNA	1.53	0.00	0.00
1183	'CLCN4'	mRNA	1.54	0.13	0.01
8970	'H2BC11'	mRNA	1.59	0.23	0.02
3592	'IL12A'	mRNA	1.62	0.20	0.01
2119	'ETV5'	mRNA	1.64	0.00	0.00
8639	'AOC3'	mRNA	1.66	0.17	0.01
81848	'SPRY4'	mRNA	1.67	0.12	0.00
4807	'NHLH1'	mRNA	1.73	0.30	0.03
8626	'TP63'	mRNA	1.74	0.06	0.00
728689	'EIF3CL'	mRNA	1.74	0.31	0.03
1846	'DUSP4'	mRNA	1.76	0.02	0.00
55509	'BATF3'	mRNA	1.80	0.35	0.04
100529239	'RPS10-NUDT3'	mRNA	1.83	0.00	0.00
6347	'CCL2'	mRNA	1.83	0.01	0.00
8507	'ENC1'	mRNA	1.90	0.14	0.01
285429	'DCAF4L1'	mRNA	1.91	0.36	0.05
7294	'TXK'	mRNA	1.94	0.33	0.04
1848	'DUSP6'	mRNA	1.97	0.00	0.00
729978	'NPIPB2'	mRNA	1.99	0.29	0.03
3491	'CCN1'	mRNA	2.01	0.00	0.00
8061	'FOSL1'	mRNA	2.05	0.00	0.00
256714	'MAP7D2'	mRNA	2.07	0.19	0.01
27063	'ANKRD1'	mRNA	2.15	0.03	0.00
83706	'FERMT3'	mRNA	2.20	0.35	0.05
23779	'ARHGAP8'	mRNA	2.24	0.29	0.03
110116772	'ZNF765-ZNF761'	mRNA	2.32	0.23	0.02

81558	'FAM117A'	mRNA	2.36	0.22	0.02
5672	'PSG4'	mRNA	2.51	0.26	0.02
3569	'IL6'	mRNA	2.51	0.00	0.00
100653515	'CEP295NL'	mRNA	2.88	0.11	0.00
100532736	'MICOS10-NBL1'	mRNA	3.05	0.18	0.01
257106	'ARHGAP30'	mRNA	3.06	0.30	0.03
10368	'CACNG3'	mRNA	3.08	0.29	0.03
93953	'GCNA'	mRNA	3.23	0.31	0.03
389396	'GLYATL3'	mRNA	3.58	0.31	0.03
148523	'CIART'	mRNA	3.69	0.29	0.03
11009	'IL24'	mRNA	3.87	0.21	0.02
6447	'SCG5'	mRNA	5.09	0.06	0.00
100529241	'HSPE1-MOB4'	mRNA	6.22	0.01	0.00
723790	'H2AC19'	mRNA	21.94	0.00	0.00

Supplementary Table S3. 93 upregulated differentially expressed genes in HepG2 cells after 48 h treatment with 2.5 μM FBX-DMEP

Gene ID	Gene Symbol	Type	log2 (FBX-DMEP /DMSO)	Qvalue (FBX-DMEP /DMSO)	Pvalue (FBX-DMEP /DMSO)
723790	'H2AC19'	mRNA	21.94	0.00	0.00
100529241	'HSPE1-MOB4'	mRNA	6.22	0.01	0.00
6447	'SCG5'	mRNA	5.09	0.06	0.00
11009	'IL24'	mRNA	3.87	0.21	0.02
148523	'CIART'	mRNA	3.69	0.29	0.03
389396	'GLYATL3'	mRNA	3.58	0.31	0.03
93953	'GCNA'	mRNA	3.23	0.31	0.03
10368	'CACNG3'	mRNA	3.08	0.29	0.03
257106	'ARHGAP30'	mRNA	3.06	0.30	0.03
100532736	'MICOS10-NBL1'	mRNA	3.05	0.18	0.01
100653515	'CEP295NL'	mRNA	2.88	0.11	0.00
3569	'IL6'	mRNA	2.51	0.00	0.00
5672	'PSG4'	mRNA	2.51	0.26	0.02
81558	'FAM117A'	mRNA	2.36	0.22	0.02
110116772	'ZNF765-ZNF761'	mRNA	2.32	0.23	0.02
23779	'ARHGAP8'	mRNA	2.24	0.29	0.03
83706	'FERMT3'	mRNA	2.20	0.35	0.05
27063	'ANKRD1'	mRNA	2.15	0.03	0.00
256714	'MAP7D2'	mRNA	2.07	0.19	0.01
8061	'FOSL1'	mRNA	2.05	0.00	0.00
3491	'CCN1'	mRNA	2.01	0.00	0.00
729978	'NPIPB2'	mRNA	1.99	0.29	0.03

1848	'DUSP6'	mRNA	1.97	0.00	0.00
7294	'TXK'	mRNA	1.94	0.33	0.04
285429	'DCAF4L1'	mRNA	1.91	0.36	0.05
8507	'ENC1'	mRNA	1.90	0.14	0.01
6347	'CCL2'	mRNA	1.83	0.01	0.00
100529239	'RPS10-NUDT3'	mRNA	1.83	0.00	0.00
55509	'BATF3'	mRNA	1.80	0.35	0.04
1846	'DUSP4'	mRNA	1.76	0.02	0.00
728689	'EIF3CL'	mRNA	1.74	0.31	0.03
8626	'TP63'	mRNA	1.74	0.06	0.00
4807	'NHLH1'	mRNA	1.73	0.30	0.03
81848	'SPRY4'	mRNA	1.67	0.12	0.00
8639	'AOC3'	mRNA	1.66	0.17	0.01
2119	'ETV5'	mRNA	1.64	0.00	0.00
3592	'IL12A'	mRNA	1.62	0.20	0.01
8970	'H2BC11'	mRNA	1.59	0.23	0.02
1183	'CLCN4'	mRNA	1.54	0.13	0.01
3017	'H2BC5'	mRNA	1.53	0.00	0.00
84959	'UBASH3B'	mRNA	1.53	0.17	0.01
220001	'VWCE'	mRNA	1.52	0.29	0.03
1164	'CKS2'	mRNA	1.49	0.06	0.00
613037	'NPIPB13'	mRNA	1.47	0.23	0.02
57393	'CLTRN'	mRNA	1.39	0.08	0.00
8676	'STX11'	mRNA	1.38	0.33	0.04
10769	'PLK2'	mRNA	1.37	0.01	0.00
114769	'CARD16'	mRNA	1.37	0.08	0.00
5923	'RASGRF1'	mRNA	1.35	0.27	0.02

199800	'ADM5'	mRNA	1.34	0.01	0.00
2354	'FOSB'	mRNA	1.33	0.02	0.00
50617	'ATP6V0A4'	mRNA	1.33	0.06	0.00
8341	'H2BC15'	mRNA	1.33	0.31	0.03
1821	'DRP2'	mRNA	1.30	0.15	0.01
22822	'PHLDA1'	mRNA	1.30	0.04	0.00
3885	'KRT34'	mRNA	1.29	0.28	0.03
8870	'IER3'	mRNA	1.28	0.00	0.00
79628	'SH3TC2'	mRNA	1.27	0.10	0.00
29091	'STXBP6'	mRNA	1.27	0.07	0.00
991	'CDC20'	mRNA	1.23	0.01	0.00
314	'AOC2'	mRNA	1.21	0.21	0.01
56256	'SERTAD4'	mRNA	1.19	0.04	0.00
1969	'EPHA2'	mRNA	1.18	0.00	0.00
51330	'TNFRSF12A'	mRNA	1.18	0.03	0.00
102800317	'TPTEP2-CSNK1E'	mRNA	1.18	0.20	0.01
100132247	'NPIPB5'	mRNA	1.17	0.01	0.00
90417	'KNSTRN'	mRNA	1.15	0.11	0.00
152519	'NIPAL1'	mRNA	1.13	0.05	0.00
26579	'MYEOV'	mRNA	1.13	0.35	0.04
375791	'CYSRT1'	mRNA	1.12	0.01	0.00
2118	'ETV4'	mRNA	1.11	0.00	0.00
10202	'DHRS2'	mRNA	1.11	0.24	0.02
4862	'NPAS2'	mRNA	1.11	0.05	0.00
2152	'F3'	mRNA	1.11	0.06	0.00
3931	'LCAT'	mRNA	1.10	0.21	0.02
652	'BMP4'	mRNA	1.10	0.08	0.00

10024	'TROAP'	mRNA	1.09	0.00	0.00
80149	'ZC3H12A'	mRNA	1.08	0.00	0.00
390	'RND3'	mRNA	1.08	0.01	0.00
55062	'WIPI1'	mRNA	1.07	0.04	0.00
6790	'AURKA'	mRNA	1.07	0.05	0.00
115362	'GBP5'	mRNA	1.06	0.11	0.00
23117	'NPIPB3'	mRNA	1.06	0.00	0.00
9531	'BAG3'	mRNA	1.05	0.04	0.00
7099	'TLR4'	mRNA	1.04	0.17	0.01
8347	'H2BC4'	mRNA	1.03	0.08	0.00
3383	'ICAM1'	mRNA	1.03	0.01	0.00
4356	'MPP3'	mRNA	1.03	0.00	0.00
2274	'FHL2'	mRNA	1.02	0.01	0.00
10627	'MYL12A'	mRNA	1.02	0.13	0.01
4281	'MID1'	mRNA	1.02	0.06	0.00
79844	'ZDHHC11'	mRNA	1.01	0.14	0.01
3229	'HOXC13'	mRNA	1.00	0.00	0.00

Supplementary Table S4. 401 downregulated differentially expressed genes in HepG2 cells after 48 h treatment with 2.5 μM FBX-DMEP.

Gene ID	Gene Symbol	Type	log2 (FBX-DMEP /DMSO)	Qvalue (FBX-DMEP /DMSO)	Pvalue (FBX-DMEP /DMSO)
100526832	'PHOSPHO2-KLHL23'	mRNA	-6.43	0.00	0.00
145781	'GCOM1'	mRNA	-5.48	0.13	0.01
4056	'LTC4S'	mRNA	-5.07	0.30	0.03
56896	'DPYSL5'	mRNA	-4.59	0.24	0.02
101059918	'GOLGA8R'	mRNA	-4.46	0.21	0.02
84767	'TRIM51'	mRNA	-4.35	0.15	0.01
221409	'SPATS1'	mRNA	-4.22	0.29	0.03
79170	'PRR15L'	mRNA	-4.22	0.07	0.00
441381	'LRRC24'	mRNA	-4.11	0.29	0.03
9032	'TM4SF5'	mRNA	-4.10	0.37	0.05
84634	'KISS1R'	mRNA	-4.09	0.35	0.04
9378	'NRXN1'	mRNA	-4.09	0.16	0.01
23768	'FLRT2'	mRNA	-4.07	0.26	0.02
780776	'TVP23A'	mRNA	-3.90	0.22	0.02
360205	'PRAC2'	mRNA	-3.79	0.33	0.04
1780	'DYNC1I1'	mRNA	-3.68	0.34	0.04
342618	'SLFN14'	mRNA	-3.39	0.23	0.02
50832	'TAS2R4'	mRNA	-3.27	0.24	0.02
643669	'CCER2'	mRNA	-3.24	0.33	0.04
388564	'TMEM238'	mRNA	-3.22	0.18	0.01
25803	'SPDEF'	mRNA	-3.18	0.06	0.00

8577	'TMEFF1'	mRNA	-3.14	0.11	0.00
1890	'TYMP'	mRNA	-3.12	0.21	0.02
8294	'H4C9'	mRNA	-2.86	0.33	0.04
63827	'BCAN'	mRNA	-2.76	0.06	0.00
2657	'GDF1'	mRNA	-2.75	0.30	0.03
100996842	'LOC100996842'	mRNA	-2.73	0.03	0.00
11309	'SLCO2B1'	mRNA	-2.63	0.35	0.04
4917	'NTN3'	mRNA	-2.61	0.17	0.01
57165	'GJC2'	mRNA	-2.61	0.14	0.01
4902	'NRTN'	mRNA	-2.58	0.08	0.00
1299	'COL9A3'	mRNA	-2.54	0.31	0.03
79927	'FAM110D'	mRNA	-2.48	0.29	0.03
79946	'C10orf95'	mRNA	-2.47	0.06	0.00
196740	'VSTM4'	mRNA	-2.47	0.28	0.03
728577	'CNTNAP3B'	mRNA	-2.46	0.09	0.00
642515	'PRRT1B'	mRNA	-2.42	0.28	0.03
140825	'NEURL2'	mRNA	-2.42	0.25	0.02
2868	'GRK4'	mRNA	-2.41	0.18	0.01
3706	'ITPKA'	mRNA	-2.40	0.02	0.00
3718	'JAK3'	mRNA	-2.39	0.26	0.02
7153	'TOP2A'	mRNA	-2.36	0.00	0.00
1137	'CHRNA4'	mRNA	-2.36	0.20	0.01
54577	'UGT1A7'	mRNA	-2.34	0.31	0.03
10023	'FRAT1'	mRNA	-2.32	0.06	0.00
83729	'INHBE'	mRNA	-2.30	0.12	0.01
9241	'NOG'	mRNA	-2.27	0.25	0.02
54492	'NEURL1B'	mRNA	-2.26	0.00	0.00

89870	'TRIM15'	mRNA	-2.26	0.27	0.03
115650	'TNFRSF13C'	mRNA	-2.24	0.11	0.00
11094	'CACFD1'	mRNA	-2.21	0.04	0.00
23204	'ARL6IP1'	mRNA	-2.18	0.00	0.00
10732	'TCFL5'	mRNA	-2.16	0.30	0.03
83259	'PCDH11Y'	mRNA	-2.14	0.31	0.03
1825	'DSC3'	mRNA	-2.13	0.22	0.02
728116	'ZBTB8B'	mRNA	-2.13	0.31	0.03
5648	'MASP1'	mRNA	-2.13	0.22	0.02
221914	'GPC2'	mRNA	-2.12	0.16	0.01
161882	'ZFPM1'	mRNA	-2.06	0.04	0.00
728492	'SERF1B'	mRNA	-2.04	0.32	0.04
55897	'MESP1'	mRNA	-2.03	0.35	0.04
646960	'PRSS56'	mRNA	-2.02	0.22	0.02
79788	'ZNF665'	mRNA	-2.00	0.32	0.04
94234	'FOXQ1'	mRNA	-1.99	0.24	0.02
90113	'VWA5B2'	mRNA	-1.99	0.30	0.03
56936	'CCDC177'	mRNA	-1.99	0.05	0.00
83481	'EPPK1'	mRNA	-1.96	0.29	0.03
401720	'FIGNL2'	mRNA	-1.95	0.09	0.00
84278	'MFSD14C'	mRNA	-1.94	0.24	0.02
1277	'COL1A1'	mRNA	-1.93	0.34	0.04
3727	'JUND'	mRNA	-1.93	0.01	0.00
100132074	'FOXO6'	mRNA	-1.92	0.11	0.00
201255	'LRRC45'	mRNA	-1.92	0.27	0.03
687	'KLF9'	mRNA	-1.92	0.18	0.01
5372	'PMM1'	mRNA	-1.91	0.37	0.05

388323	'GLTPD2'	mRNA	-1.88	0.29	0.03
6752	'SSTR2'	mRNA	-1.86	0.20	0.01
253559	'CADM2'	mRNA	-1.86	0.36	0.05
84968	'PNMA6A'	mRNA	-1.85	0.17	0.01
79782	'LRRC31'	mRNA	-1.83	0.03	0.00
389692	'MAFA'	mRNA	-1.81	0.28	0.03
1628	'DBP'	mRNA	-1.81	0.06	0.00
155382	'VPS37D'	mRNA	-1.79	0.33	0.04
389816	'LRRC26'	mRNA	-1.79	0.03	0.00
199699	'DAND5'	mRNA	-1.79	0.31	0.03
23101	'MCF2L2'	mRNA	-1.78	0.22	0.02
3795	'KHK'	mRNA	-1.78	0.02	0.00
79842	'ZBTB3'	mRNA	-1.77	0.01	0.00
5334	'PLCL1'	mRNA	-1.77	0.01	0.00
90427	'BMF'	mRNA	-1.75	0.05	0.00
1028	'CDKN1C'	mRNA	-1.75	0.01	0.00
25794	'FSCN2'	mRNA	-1.74	0.28	0.03
79632	'FAM184A'	mRNA	-1.73	0.30	0.03
91828	'EXOC3L4'	mRNA	-1.72	0.29	0.03
10675	'CSPG5'	mRNA	-1.72	0.00	0.00
79772	'MCTP1'	mRNA	-1.71	0.02	0.00
255783	'INAFM1'	mRNA	-1.71	0.21	0.02
55450	'CAMK2N1'	mRNA	-1.70	0.03	0.00
53342	'IL17D'	mRNA	-1.69	0.26	0.02
983	'CDK1'	mRNA	-1.68	0.00	0.00
643680	'MS4A4E'	mRNA	-1.68	0.31	0.03
4130	'MAP1A'	mRNA	-1.67	0.00	0.00

84922	'FIZ1'	mRNA	-1.67	0.01	0.00
2306	'FOXD2'	mRNA	-1.67	0.22	0.02
155185	'AMZ1'	mRNA	-1.66	0.03	0.00
11065	'UBE2C'	mRNA	-1.66	0.00	0.00
353376	'TICAM2'	mRNA	-1.65	0.30	0.03
83882	'TSPAN10'	mRNA	-1.65	0.20	0.01
3110	'MNX1'	mRNA	-1.64	0.17	0.01
255374	'MBLAC1'	mRNA	-1.63	0.34	0.04
6094	'ROM1'	mRNA	-1.63	0.32	0.04
254552	'NUDT8'	mRNA	-1.63	0.17	0.01
379	'ARL4D'	mRNA	-1.60	0.04	0.00
401097	'C3orf80'	mRNA	-1.59	0.11	0.00
57622	'LRFN1'	mRNA	-1.59	0.00	0.00
91179	'SCARF2'	mRNA	-1.59	0.18	0.01
100507588	'TGFBR3L'	mRNA	-1.58	0.13	0.01
55512	'SMPD3'	mRNA	-1.57	0.32	0.04
79002	'TRIR'	mRNA	-1.57	0.01	0.00
90673	'PPP1R3E'	mRNA	-1.57	0.08	0.00
135152	'B3GAT2'	mRNA	-1.56	0.32	0.04
257	'ALX3'	mRNA	-1.55	0.01	0.00
376497	'SLC27A1'	mRNA	-1.54	0.02	0.00
162494	'RHBDL3'	mRNA	-1.54	0.15	0.01
8364	'H4C3'	mRNA	-1.53	0.25	0.02
114783	'LMTK3'	mRNA	-1.53	0.24	0.02
388403	'YPEL2'	mRNA	-1.53	0.01	0.00
388963	'C2orf81'	mRNA	-1.53	0.23	0.02
6659	'SOX4'	mRNA	-1.53	0.00	0.00

56413	'LTB4R2'	mRNA	-1.52	0.18	0.01
93145	'OLFM2'	mRNA	-1.50	0.22	0.02
23232	'TBC1D12'	mRNA	-1.50	0.01	0.00
147687	'ZNF417'	mRNA	-1.49	0.03	0.00
27022	'FOXD3'	mRNA	-1.48	0.33	0.04
219699	'UNC5B'	mRNA	-1.48	0.02	0.00
80352	'RNF39'	mRNA	-1.48	0.27	0.02
3903	'LAIR1'	mRNA	-1.46	0.21	0.02
340152	'ZC3H12D'	mRNA	-1.45	0.29	0.03
200312	'RNF215'	mRNA	-1.45	0.21	0.02
64599	'GIGYF1'	mRNA	-1.45	0.01	0.00
79447	'PAGR1'	mRNA	-1.45	0.18	0.01
374920	'ZSWIM9'	mRNA	-1.44	0.22	0.02
7475	'WNT6'	mRNA	-1.44	0.23	0.02
220213	'OTUD1'	mRNA	-1.43	0.13	0.01
648791	'PPP1R3G'	mRNA	-1.43	0.13	0.01
110806298	'C17orf113'	mRNA	-1.42	0.24	0.02
51222	'ZNF219'	mRNA	-1.42	0.19	0.01
29775	'CARD10'	mRNA	-1.42	0.04	0.00
387787	'LIPT2'	mRNA	-1.40	0.17	0.01
7465	'WEE1'	mRNA	-1.40	0.00	0.00
5106	'PCK2'	mRNA	-1.40	0.20	0.01
10451	'VAV3'	mRNA	-1.39	0.00	0.00
117247	'SLC16A10'	mRNA	-1.39	0.17	0.01
90007	'MIDN'	mRNA	-1.39	0.00	0.00
7161	'TP73'	mRNA	-1.39	0.18	0.01
9028	'RHBDL1'	mRNA	-1.38	0.24	0.02

158055	'C9orf163'	mRNA	-1.38	0.28	0.03
58487	'CREBZF'	mRNA	-1.38	0.04	0.00
148252	'DIRAS1'	mRNA	-1.38	0.27	0.02
57134	'MAN1C1'	mRNA	-1.38	0.16	0.01
55147	'RBM23'	mRNA	-1.38	0.00	0.00
3838	'KPNA2'	mRNA	-1.38	0.02	0.00
63920	'ZBED8'	mRNA	-1.38	0.16	0.01
50509	'COL5A3'	mRNA	-1.37	0.33	0.04
5507	'PPP1R3C'	mRNA	-1.37	0.04	0.00
3868	'KRT16'	mRNA	-1.37	0.19	0.01
7018	'TF'	mRNA	-1.37	0.17	0.01
118460	'EXOSC6'	mRNA	-1.36	0.14	0.01
7428	'VHL'	mRNA	-1.36	0.17	0.01
5296	'PIK3R2'	mRNA	-1.36	0.00	0.00
3619	'INCENP'	mRNA	-1.36	0.00	0.00
2297	'FOXD1'	mRNA	-1.35	0.22	0.02
55374	'TMCO6'	mRNA	-1.34	0.21	0.01
9783	'RIMS3'	mRNA	-1.34	0.22	0.02
284023	'RNF227'	mRNA	-1.34	0.00	0.00
7049	'TGFBR3'	mRNA	-1.33	0.00	0.00
9435	'CHST2'	mRNA	-1.33	0.28	0.03
125170	'MIEF2'	mRNA	-1.33	0.10	0.00
10290	'SPEG'	mRNA	-1.33	0.10	0.00
54910	'SEMA4C'	mRNA	-1.32	0.06	0.00
283208	'P4HA3'	mRNA	-1.32	0.18	0.01
55344	'PLCXD1'	mRNA	-1.32	0.00	0.00
79798	'ARMC5'	mRNA	-1.32	0.21	0.01

92815	'H2AW'	mRNA	-1.31	0.37	0.05
55561	'CDC42BPG'	mRNA	-1.31	0.12	0.01
84705	'GTPBP3'	mRNA	-1.31	0.01	0.00
57547	'ZNF624'	mRNA	-1.31	0.21	0.01
23467	'NPTXR'	mRNA	-1.31	0.00	0.00
23385	'NCSTN'	mRNA	-1.31	0.01	0.00
899	'CCNF'	mRNA	-1.31	0.00	0.00
126133	'ALDH16A1'	mRNA	-1.31	0.15	0.01
57010	'CABP4'	mRNA	-1.31	0.17	0.01
57716	'PRX'	mRNA	-1.30	0.28	0.03
23178	'PASK'	mRNA	-1.30	0.01	0.00
84264	'HAGHL'	mRNA	-1.29	0.28	0.03
84932	'RAB2B'	mRNA	-1.29	0.00	0.00
4092	'SMAD7'	mRNA	-1.29	0.00	0.00
9826	'ARHGEF11'	mRNA	-1.29	0.00	0.00
349075	'ZNF713'	mRNA	-1.29	0.07	0.00
1870	'E2F2'	mRNA	-1.28	0.00	0.00
8325	'FZD8'	mRNA	-1.28	0.22	0.02
84787	'KMT5C'	mRNA	-1.28	0.05	0.00
8646	'CHRD'	mRNA	-1.28	0.26	0.02
4082	'MARCKS'	mRNA	-1.27	0.18	0.01
3216	'HOXB6'	mRNA	-1.27	0.28	0.03
89849	'ATG16L2'	mRNA	-1.27	0.21	0.02
11264	'PXMP4'	mRNA	-1.27	0.28	0.03
84915	'FAM222A'	mRNA	-1.27	0.11	0.00
2116	'ETV2'	mRNA	-1.26	0.35	0.04
2178	'FANCE'	mRNA	-1.26	0.07	0.00

389333	'PROB1'	mRNA	-1.25	0.28	0.03
57643	'ZSWIM5'	mRNA	-1.25	0.00	0.00
401265	'KLHL31'	mRNA	-1.25	0.26	0.02
1869	'E2F1'	mRNA	-1.25	0.01	0.00
9696	'CROCC'	mRNA	-1.25	0.26	0.02
138428	'PTRH1'	mRNA	-1.25	0.31	0.03
113115	'MTFR2'	mRNA	-1.25	0.05	0.00
90353	'CTU1'	mRNA	-1.25	0.35	0.04
54876	'DCAF16'	mRNA	-1.24	0.00	0.00
4162	'MCAM'	mRNA	-1.24	0.00	0.00
26271	'FBXO5'	mRNA	-1.24	0.00	0.00
359948	'IRF2BP2'	mRNA	-1.24	0.17	0.01
414919	'C8orf82'	mRNA	-1.23	0.31	0.03
285521	'COX18'	mRNA	-1.23	0.16	0.01
6456	'SH3GL2'	mRNA	-1.23	0.01	0.00
84875	'PARP10'	mRNA	-1.23	0.18	0.01
144699	'FBXL14'	mRNA	-1.23	0.16	0.01
3303	'HSPA1A'	mRNA	-1.23	0.09	0.00
10181	'RBM5'	mRNA	-1.23	0.00	0.00
388552	'BLOC1S3'	mRNA	-1.22	0.06	0.00
8535	'CBX4'	mRNA	-1.22	0.06	0.00
580	'BARD1'	mRNA	-1.22	0.00	0.00
10251	'SPRY3'	mRNA	-1.22	0.30	0.03
29904	'EEF2K'	mRNA	-1.21	0.00	0.00
2319	'FLOT2'	mRNA	-1.21	0.00	0.00
1316	'KLF6'	mRNA	-1.20	0.00	0.00
9592	'IER2'	mRNA	-1.20	0.31	0.03

2621	'GAS6'	mRNA	-1.20	0.22	0.02
100533105	'C8orf44-SGK3'	mRNA	-1.20	0.35	0.05
116028	'RMI2'	mRNA	-1.20	0.08	0.00
7374	'UNG'	mRNA	-1.19	0.00	0.00
114822	'RHPN1'	mRNA	-1.19	0.29	0.03
79037	'PVRIG'	mRNA	-1.19	0.35	0.04
57479	'PRR12'	mRNA	-1.19	0.00	0.00
1943	'EFNA2'	mRNA	-1.19	0.27	0.03
29968	'PSAT1'	mRNA	-1.18	0.00	0.00
90576	'ZNF799'	mRNA	-1.18	0.32	0.04
2030	'SLC29A1'	mRNA	-1.18	0.01	0.00
3209	'HOXA13'	mRNA	-1.18	0.00	0.00
41	'ASIC1'	mRNA	-1.18	0.00	0.00
254013	'ETFBKMT'	mRNA	-1.18	0.34	0.04
144455	'E2F7'	mRNA	-1.17	0.00	0.00
22859	'ADGRL1'	mRNA	-1.17	0.01	0.00
9451	'EIF2AK3'	mRNA	-1.17	0.00	0.00
9355	'LHX2'	mRNA	-1.17	0.21	0.01
399664	'MEX3D'	mRNA	-1.17	0.32	0.04
10347	'ABCA7'	mRNA	-1.17	0.31	0.03
729438	'CASTOR2'	mRNA	-1.17	0.02	0.00
248	'ALPI'	mRNA	-1.17	0.15	0.01
79803	'HPS6'	mRNA	-1.16	0.06	0.00
51337	'THEM6'	mRNA	-1.16	0.18	0.01
23456	'ABCB10'	mRNA	-1.16	0.11	0.00
340719	'NANOS1'	mRNA	-1.16	0.33	0.04
100131244	'ANKRD63'	mRNA	-1.16	0.35	0.04

1808	'DPYSL2'	mRNA	-1.16	0.00	0.00
5190	'PEX6'	mRNA	-1.16	0.30	0.03
126272	'EID2B'	mRNA	-1.16	0.32	0.04
23211	'ZC3H4'	mRNA	-1.15	0.00	0.00
51043	'ZBTB7B'	mRNA	-1.15	0.21	0.01
25840	'METTL7A'	mRNA	-1.15	0.15	0.01
2634	'GBP2'	mRNA	-1.14	0.28	0.03
9837	'GINS1'	mRNA	-1.14	0.00	0.00
29946	'SERTAD3'	mRNA	-1.14	0.00	0.00
5604	'MAP2K1'	mRNA	-1.14	0.30	0.03
374654	'KIF7'	mRNA	-1.14	0.17	0.01
51005	'AMDHD2'	mRNA	-1.13	0.27	0.02
100381270	'ZBED6'	mRNA	-1.13	0.00	0.00
128272	'ARHGEF19'	mRNA	-1.13	0.28	0.03
10882	'C1QL1'	mRNA	-1.13	0.34	0.04
9422	'ZNF264'	mRNA	-1.13	0.01	0.00
55160	'ARHGEF10L'	mRNA	-1.13	0.17	0.01
63971	'KIF13A'	mRNA	-1.13	0.00	0.00
140545	'RNF32'	mRNA	-1.13	0.33	0.04
100293516	'ZNF587B'	mRNA	-1.13	0.01	0.00
439921	'MXRA7'	mRNA	-1.13	0.34	0.04
80235	'PIGZ'	mRNA	-1.13	0.18	0.01
9894	'TELO2'	mRNA	-1.13	0.19	0.01
901	'CCNG2'	mRNA	-1.12	0.10	0.00
8425	'LTBP4'	mRNA	-1.12	0.25	0.02
10965	'ACOT2'	mRNA	-1.12	0.32	0.04
8877	'SPHK1'	mRNA	-1.12	0.28	0.03

7138	'TNNT1'	mRNA	-1.12	0.13	0.01
382	'ARF6'	mRNA	-1.12	0.07	0.00
151903	'CCDC12'	mRNA	-1.12	0.03	0.00
2021	'ENDOG'	mRNA	-1.12	0.37	0.05
727910	'TLCD2'	mRNA	-1.12	0.34	0.04
51282	'SCAND1'	mRNA	-1.12	0.34	0.04
5933	'RBL1'	mRNA	-1.11	0.00	0.00
10763	'NES'	mRNA	-1.11	0.07	0.00
201266	'SLC39A11'	mRNA	-1.11	0.13	0.01
6666	'SOX12'	mRNA	-1.11	0.31	0.03
26000	'TBC1D10B'	mRNA	-1.11	0.05	0.00
84687	'PPP1R9B'	mRNA	-1.11	0.02	0.00
8693	'GALNT4'	mRNA	-1.10	0.26	0.02
727800	'RNF208'	mRNA	-1.10	0.35	0.04
2188	'FANCF'	mRNA	-1.10	0.09	0.00
254102	'EHBP1L1'	mRNA	-1.10	0.24	0.02
54345	'SOX18'	mRNA	-1.10	0.32	0.04
4131	'MAP1B'	mRNA	-1.10	0.00	0.00
6751	'SSTR1'	mRNA	-1.10	0.00	0.00
284058	'KANSL1'	mRNA	-1.10	0.00	0.00
23401	'FRAT2'	mRNA	-1.10	0.30	0.03
26048	'ZNF500'	mRNA	-1.10	0.03	0.00
284004	'HEXD'	mRNA	-1.10	0.25	0.02
79873	'NUDT18'	mRNA	-1.10	0.34	0.04
8743	'TNFSF10'	mRNA	-1.09	0.25	0.02
573	'BAG1'	mRNA	-1.09	0.28	0.03
286257	'PAXX'	mRNA	-1.09	0.31	0.03

9672	'SDC3'	mRNA	-1.09	0.00	0.00
92979	'MARCHF9'	mRNA	-1.09	0.29	0.03
642475	'MROH6'	mRNA	-1.09	0.29	0.03
94122	'SYTL5'	mRNA	-1.08	0.31	0.03
10279	'PRSS16'	mRNA	-1.08	0.13	0.01
166824	'RASSF6'	mRNA	-1.08	0.29	0.03
151230	'KLHL23'	mRNA	-1.08	0.01	0.00
8771	'TNFRSF6B'	mRNA	-1.08	0.26	0.02
55367	'PIDD1'	mRNA	-1.08	0.19	0.01
898	'CCNE1'	mRNA	-1.08	0.00	0.00
90378	'SAMD1'	mRNA	-1.08	0.00	0.00
334	'APLP2'	mRNA	-1.08	0.00	0.00
6494	'SIPA1'	mRNA	-1.08	0.19	0.01
3217	'HOXB7'	mRNA	-1.08	0.05	0.00
56937	'PMEPA1'	mRNA	-1.07	0.00	0.00
1747	'DLX3'	mRNA	-1.07	0.07	0.00
29057	'FAM156A'	mRNA	-1.07	0.03	0.00
101928841	'LOC101928841'	mRNA	-1.07	0.21	0.02
10541	'ANP32B'	mRNA	-1.07	0.32	0.04
284069	'FAM171A2'	mRNA	-1.07	0.34	0.04
54058	'C21orf58'	mRNA	-1.07	0.14	0.01
9134	'CCNE2'	mRNA	-1.06	0.08	0.00
23468	'CBX5'	mRNA	-1.06	0.01	0.00
8408	'ULK1'	mRNA	-1.06	0.31	0.03
92092	'ZC3HAV1L'	mRNA	-1.06	0.20	0.01
54487	'DGCR8'	mRNA	-1.06	0.00	0.00
388228	'SBK1'	mRNA	-1.06	0.34	0.04

101060684	'NBPF26'	mRNA	-1.06	0.24	0.02
2109	'ETFB'	mRNA	-1.06	0.35	0.04
5413	'SEPTIN5'	mRNA	-1.06	0.10	0.00
81621	'KAZALD1'	mRNA	-1.06	0.21	0.02
1060	'CENPC'	mRNA	-1.06	0.04	0.00
79191	'IRX3'	mRNA	-1.06	0.23	0.02
79713	'IGFLR1'	mRNA	-1.05	0.13	0.01
10607	'TBL3'	mRNA	-1.05	0.21	0.02
338879	'RNASE10'	mRNA	-1.05	0.31	0.03
6941	'TCF19'	mRNA	-1.05	0.00	0.00
91748	'MIDEAS'	mRNA	-1.05	0.00	0.00
113146	'AHNAK2'	mRNA	-1.05	0.00	0.00
22920	'KIFAP3'	mRNA	-1.05	0.00	0.00
126792	'B3GALT6'	mRNA	-1.05	0.22	0.02
2444	'FRK'	mRNA	-1.05	0.06	0.00
727	'C5'	mRNA	-1.05	0.13	0.01
550631	'CCDC157'	mRNA	-1.04	0.26	0.02
116225	'ZMYND19'	mRNA	-1.04	0.35	0.04
55027	'HEATR3'	mRNA	-1.04	0.00	0.00
2619	'GAS1'	mRNA	-1.04	0.25	0.02
9781	'RNF144A'	mRNA	-1.04	0.21	0.02
84936	'ZFYVE19'	mRNA	-1.04	0.34	0.04
91433	'RCCD1'	mRNA	-1.04	0.22	0.02
4241	'MELTF'	mRNA	-1.04	0.23	0.02
10536	'P3H3'	mRNA	-1.04	0.37	0.05
84061	'MAGT1'	mRNA	-1.04	0.28	0.03
85451	'UNK'	mRNA	-1.04	0.00	0.00

6614	'SIGLEC1'	mRNA	-1.04	0.31	0.03
4172	'MCM3'	mRNA	-1.03	0.01	0.00
29781	'NCAPH2'	mRNA	-1.03	0.27	0.02
8208	'CHAF1B'	mRNA	-1.03	0.00	0.00
107983993	'UPK3BL2'	mRNA	-1.03	0.35	0.04
1852	'DUSP9'	mRNA	-1.03	0.33	0.04
390980	'ZNF805'	mRNA	-1.03	0.21	0.01
390874	'ONECUT3'	mRNA	-1.03	0.09	0.00
954	'ENTPD2'	mRNA	-1.02	0.23	0.02
56654	'NPDC1'	mRNA	-1.02	0.35	0.04
387509	'GPR153'	mRNA	-1.02	0.18	0.01
65988	'ZNF747'	mRNA	-1.02	0.05	0.00
283008	'NUTM2E'	mRNA	-1.02	0.37	0.05
79812	'MMRN2'	mRNA	-1.02	0.31	0.03
23403	'FBXO46'	mRNA	-1.02	0.03	0.00
8799	'PEX11B'	mRNA	-1.02	0.06	0.00
114787	'GPRIN1'	mRNA	-1.02	0.00	0.00
783	'CACNB2'	mRNA	-1.02	0.10	0.00
79966	'SCD5'	mRNA	-1.02	0.07	0.00
768211	'RELL1'	mRNA	-1.01	0.10	0.00
55719	'SLF2'	mRNA	-1.01	0.00	0.00
2837	'UTS2R'	mRNA	-1.01	0.30	0.03
348487	'FAM131C'	mRNA	-1.01	0.18	0.01
643988	'FNDC10'	mRNA	-1.01	0.10	0.00
3169	'FOXA1'	mRNA	-1.00	0.00	0.00
200205	'IBA57'	mRNA	-1.00	0.13	0.01
30846	'EHD2'	mRNA	-1.00	0.00	0.00

93185	'IGSF8'	mRNA	-1.00	0.28	0.03
1609	'DGKQ'	mRNA	-1.00	0.34	0.04

Supplementary Table S5. CMap analysis reveals that FBX-DMEP treatment induces a gene expression profile similar to that of microtubule inhibitors. 2.5 μM FBX-DMEP treatment for 48 hours in HepG2 cells induces a gene expression pattern comparable to known microtubule inhibitors, with 11 out of the top 20 hit compounds targeting tubulin

Score	Name	Description
99.58	MAZ-51	VEGFR inhibitor
99.44	prostratin	PKC activator
99.33	SKF-96365	Calcium channel blocker
99.33	chelidonine	Tubulin inhibitor
99.12	ingenol	PKC activator
99.01	HU-211	Glutamate receptor antagonist
98.94	YK-4279	Apoptosis stimulant
98.91	fenbendazole	Tubulin inhibitor
98.84	mebendazole	Tubulin inhibitor
98.8	flubendazole	Tubulin inhibitor
98.8	albendazole	Anthelmintic
98.77	vindesine	Tubulin inhibitor
98.77	amonafide	Topoisomerase inhibitor
98.77	oxibendazole	Tubulin inhibitor
98.77	nocodazole	Tubulin inhibitor
98.73	KF-38789	P-selectin inhibitor
98.73	ABT-751	Tubulin inhibitor
98.63	vincristine	Tubulin inhibitor
98.41	vinblastine	Microtubule inhibitor
98.38	diphenyleneiodonium	Nitric oxide synthase inhibitor

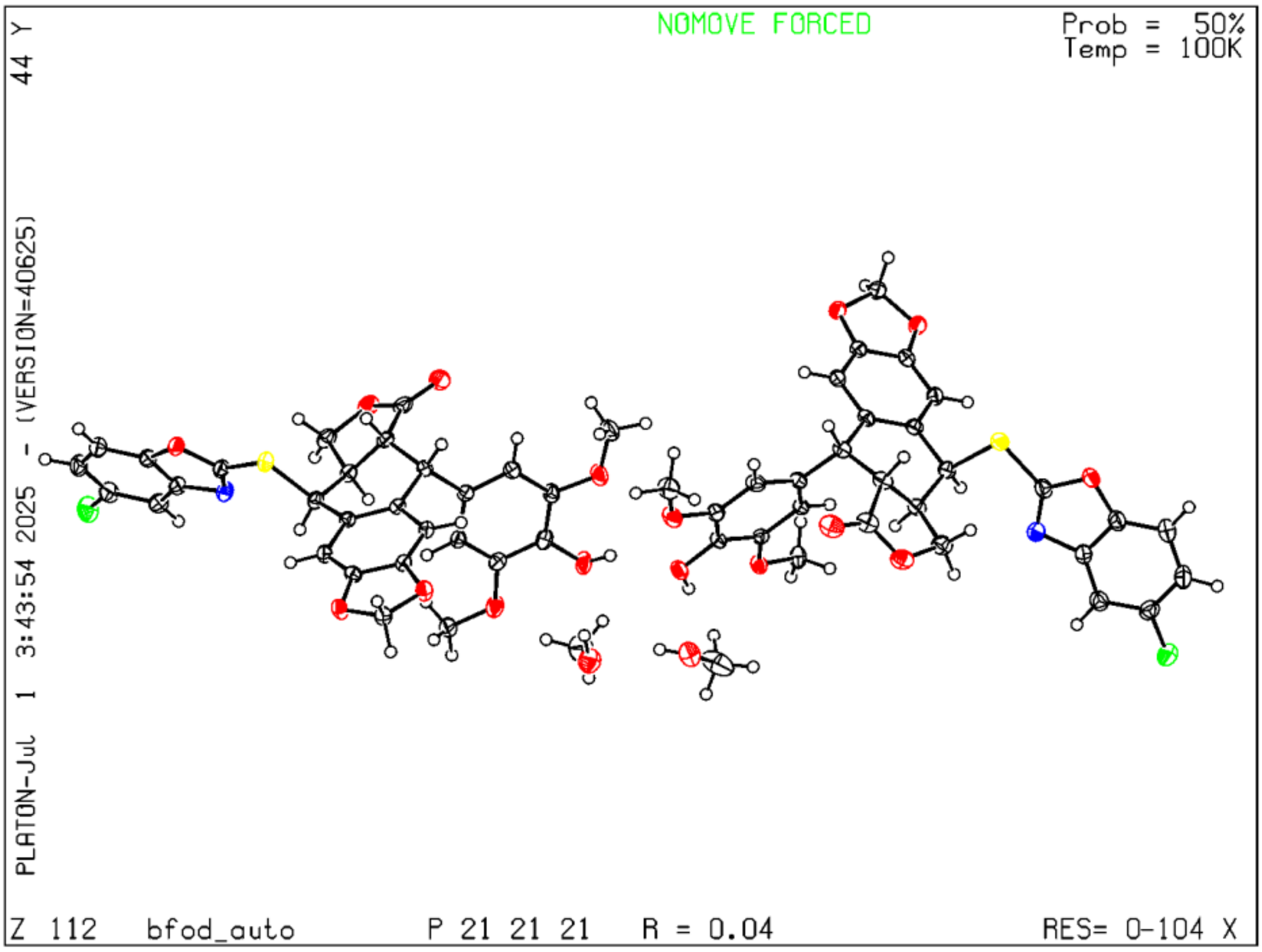

Supplementary Figure S1. X-ray crystal structure of FBX-DMEP (CCDC 2466319).

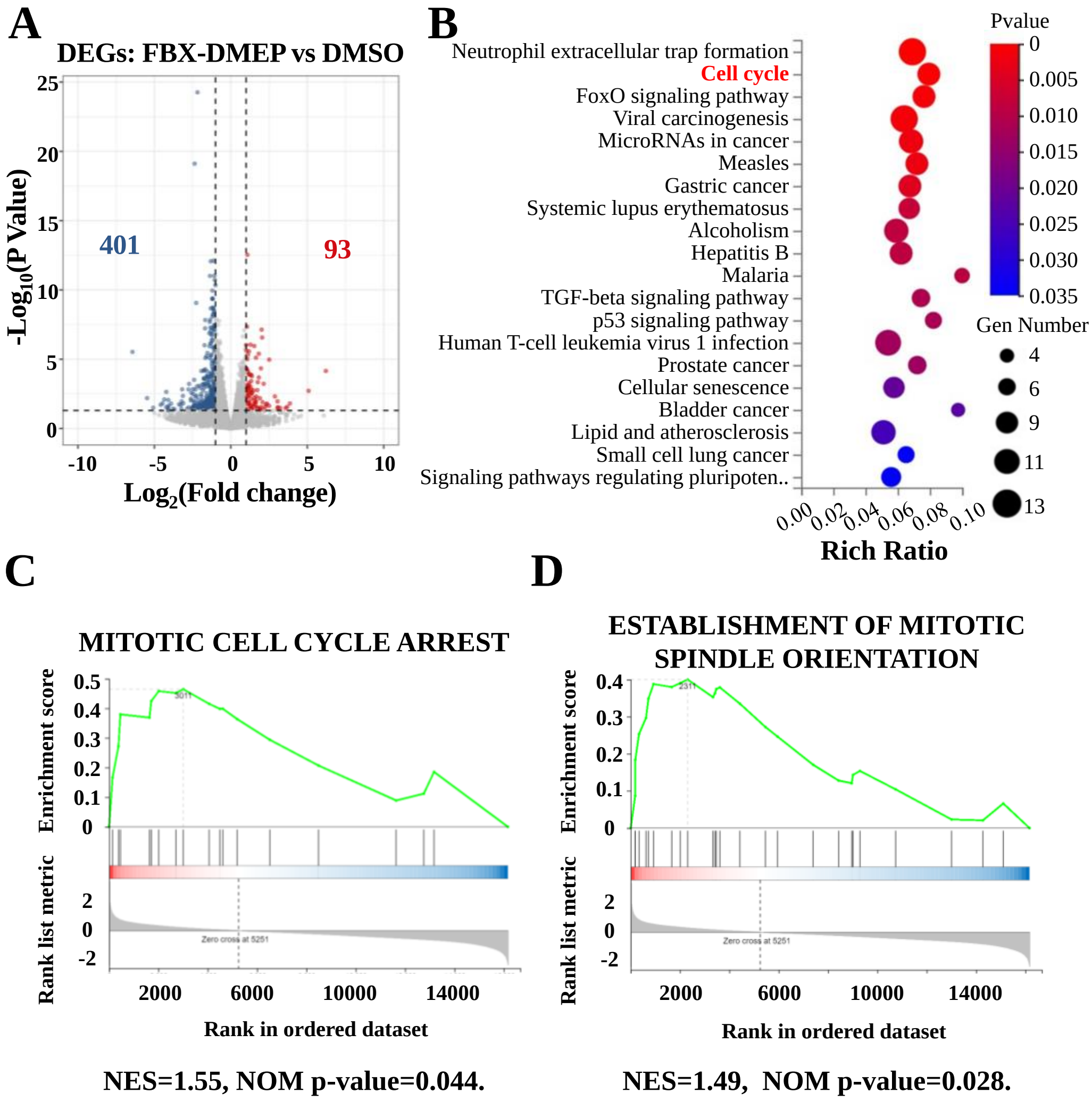

Supplementary Figure S2. Global transcriptomic responses of HepG2 cells following FBX-DMEP treatment. (A) Volcano plot showing differentially expressed genes (DEGs) identified by RNA-seq analysis between FBX-DMEP–treated and control HepG2 cells. (B) KEGG pathway enrichment analysis of differentially expressed genes (fold change ⩾ 2, $P < 0.05$) from RNA-seq of HepG2 cells treated with FBX-DMEP (10 μM) versus DMSO control for 24 h ($n = 3$ independent samples per group). Cell cycle-related pathways are highlighted. (C–D) Gene Set Enrichment Analysis (GSEA) of mitosis-related biological processes: (C) Enrichment plot of the gene set GOBP MITOTIC CELL CYCLE ARREST. (D) Enrichment plot of the gene set GOBP ESTABLISHMENT OF MITOTIC SPINDLE ORIENTATION.

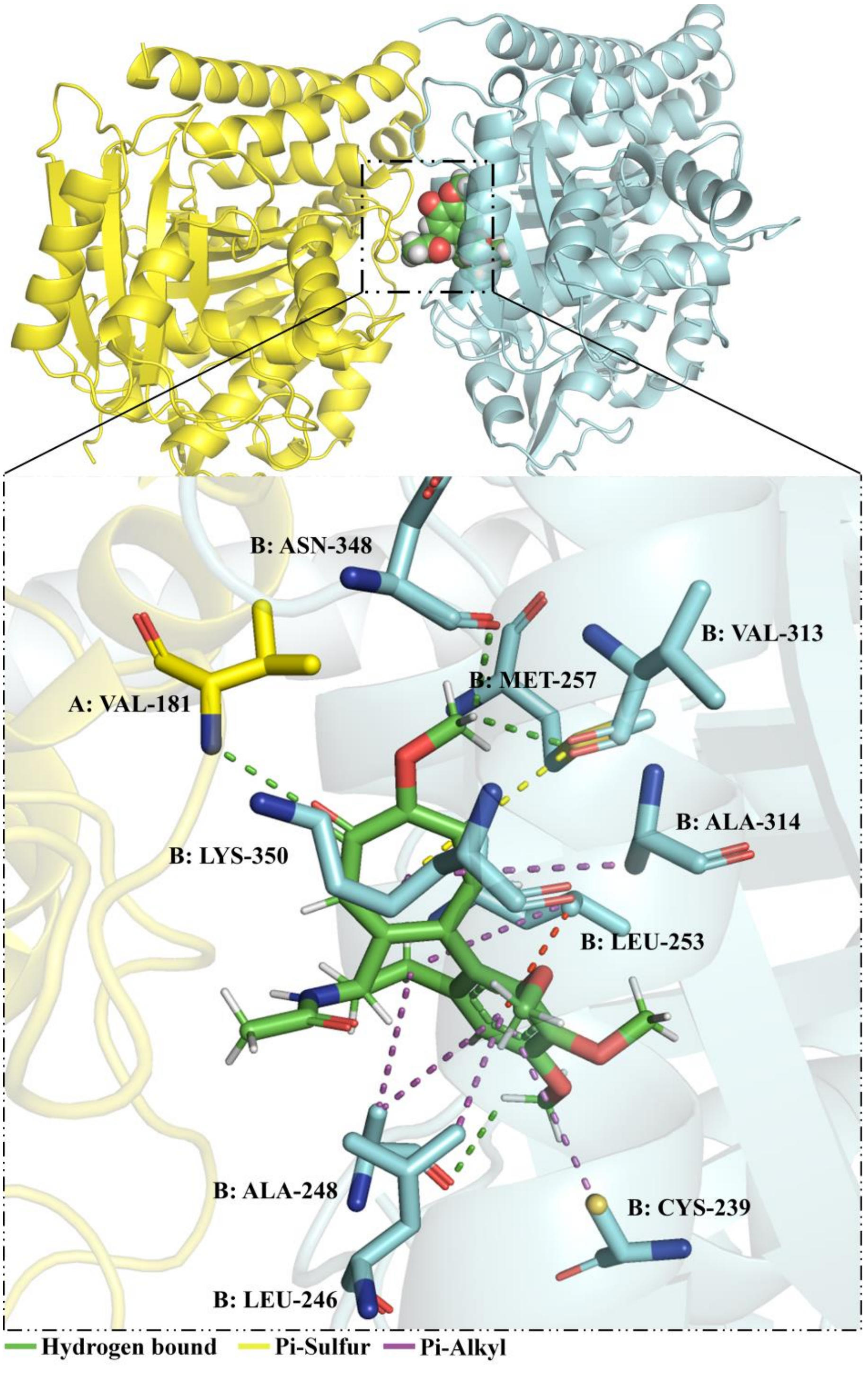

Supplementary Figure S3. Colchicine interaction with tubulin. Docking simulation of Colchicine within the colchicine-binding pocket (PDB: 6XER) of the α- (yellow) and β-tubulin (blue) heterodimer, illustrating key contacts with critical residues.

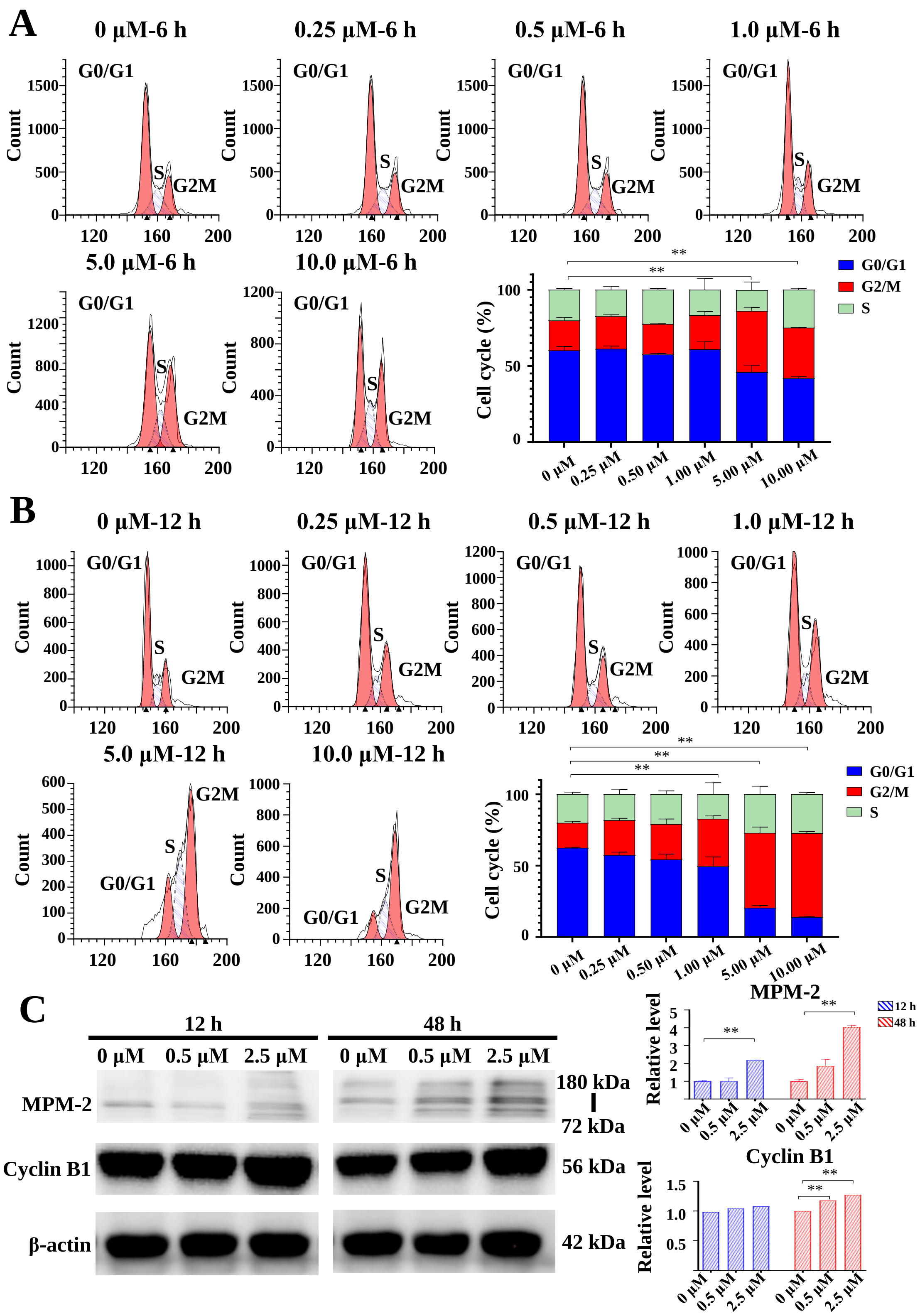

Supplementary Figure S4. FBX-DMEP induces G_2/M cell cycle arrest in HepG2 cells. Cell cycle analysis of HepG2 cells were treated with 0.25, 0.5, 1.0, 5.0 and 10 μM FBX-DMEP or DMSO for 6 (A) or (B) 12 hours. ***P* < 0.01 compared with vehicle control at 6 h and 12 h.

(C). Immunoblotting and quantitative of MPM-2 and Cyclin B1. β-actin was used as a loading control (n = 3; P values determined by one-way ANOVA). Compared with control treatment (0 μM FBX-DMEP), 2.5 μM FBX-DMEP significantly increased MPM-2 expression at 12 h (P = 0.007) and 48 h (P = 0.0001). Cyclin B1 expression was also significantly increased by 0.5 μM (P = 0.0197) and 2.5 μM (P = 0.0027) FBX-DMEP at 48 h. Data are presented as mean ± SEM. **$P < 0.01$ compared with vehicle control at 12 h and 48 h.

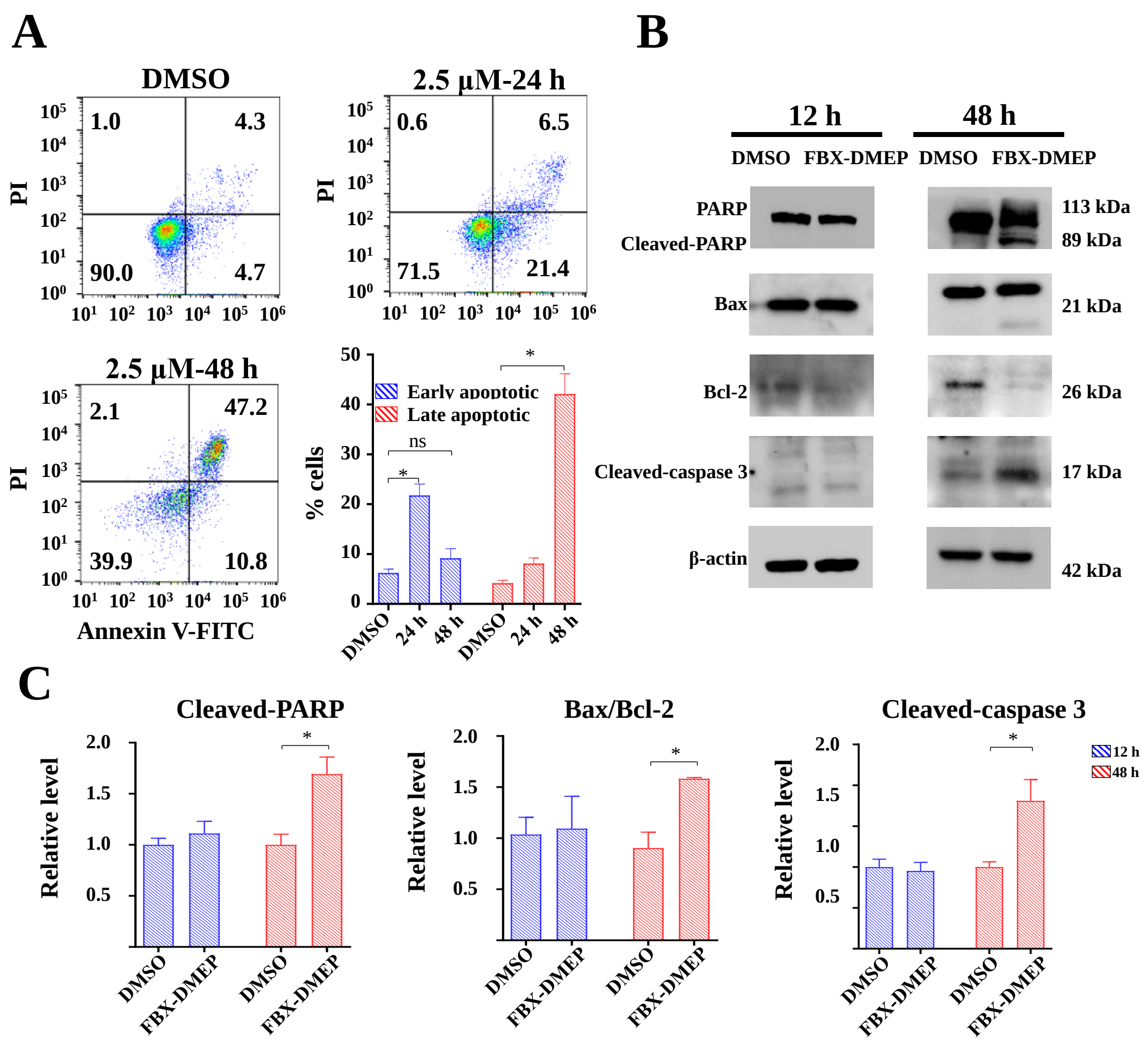

Supplementary Figure S5. FBX-DMEP induces apoptosis in HepG2 cells. (A) Flow cytometry analysis of time-dependent induction of apoptosis by FBX-DMEP (2.5 μM) at 0, 24, and 48 h. Annexin V-positive/PI-negative cells were defined as early apoptotic cells, whereas Annexin V-positive/PI-positive cells were defined as late apoptotic cells. ns = not significant, *$P < 0.05$ versus early apoptotic cells in the vehicle control group; *$P < 0.05$ versus late apoptotic cells in the vehicle control group (n = 3 per group; one-way ANOVA). Compared with control treatment (0 μM FBX-DMEP), 2.5 μM FBX-DMEP significantly increased early apoptosis at 24 h ($P = 0.0014$), while early apoptosis at 48 h was not significantly altered ($P = 0.4363$). Late apoptosis was not significantly affected at 24 h ($P = 0.4624$) but was markedly increased at 48 h ($P < 0.0001$). Representative immunoblots (B) and quantification (C) of cleaved PARP, Bax, Bcl-2, and cleaved caspase-3 in HepG2 cells treated with FBX-DMEP (2.5 μM) for 48 h (n = 3 per group; unpaired two-tailed Student's t-test). Compared with vehicle

control, FBX-DMEP significantly increased cleaved PARP ($P = 0.0242$), the Bax/Bcl-2 ratio ($P = 0.0120$), and cleaved caspase-3 ($P = 0.0392$). Data are presented as mean ± SEM. *$P < 0.05$ versus vehicle control at 48 h.
